# Towards a critical evaluation of an empirical and volume-based solvation function for ligand docking

**DOI:** 10.1371/journal.pone.0174336

**Published:** 2017-03-21

**Authors:** Heloisa S. Muniz, Alessandro S. Nascimento

**Affiliations:** Instituto de Física de São Carlos. Av. Trabalhador São-Carlense, 400. Centro São Carlos, SP, Brazil; University of Lincoln, UNITED KINGDOM

## Abstract

Molecular docking is an important tool for the discovery of new biologically active molecules given that the receptor structure is known. An excellent environment for the development of new methods and improvement of the current methods is being provided by the rapid growth in the number of proteins with known structure. The evaluation of the solvation energies outstands among the challenges for the modeling of the receptor-ligand interactions, especially in the context of molecular docking where a fast, though accurate, evaluation is ought to be achieved. Here we evaluated a variation of the desolvation energy model proposed by Stouten (Stouten P.F.W. et al, Molecular Simulation, 1993, 10: 97–120), or SV model. The SV model showed a linear correlation with experimentally determined solvation energies, as available in the database FreeSolv. However, when used in retrospective docking simulations using the benchmarks DUD, charged-matched DUD and DUD-Enhanced, the SV model resulted in poorer enrichments when compared to a pure force field model with no correction for solvation effects. The data provided here is consistent with other empirical solvation models employed in the context of molecular docking and indicates that a good model to account for solvent effects is still a goal to achieve. On the other hand, despite the inability to improve the enrichment of retrospective simulations, the SV solvation model showed an interesting ability to reduce the number of molecules with net charge -2 and -3 *e* among the top-scored molecules in a prospective test.

## Introduction

The increased number of solved protein structures provide a unique opportunity for the application of the so-called ‘structure-based’ methods for the development of new chemical entities able to regulate biological systems [1]. Additionally, since many structures have ligands bound to macromolecules, about 73% according to a naïve search in the PDB website [2], there is a favorable situation for the development of novel approaches and optimization of the current models for protein-ligand interaction [3,4].

The thermodynamics of ligand binding is based on the equilibrium between a receptor R and a ligand L, forming a complex RL: *R* + *L* ↔ *RL*. The most important thermodynamic quantity associated with the measurement of this interaction is the change in the free energy of the system [5]. This quantity can be evaluated using computationally expensive methods such as free energy perturbation (FEP) or thermodynamic integration (TI) [6]. On the other hand, inexpensive methods are available nowadays to rapidly score molecular interactions. In this scenario, the potential energies associated to binding of a ligand can be readily computed and used as an indicator of the strength of binding.

One of the most employed models for scoring protein-ligand interactions in terms of molecular modeling involves the use of an interaction potential energy as computed by force fields such as CHARMM, AMBER, OPLS or GROMOS [7], among others. For a rigid receptor, the interaction energy is typically reduced to a Lennard-Jones potential, modeling the van der Waals interaction among ligand and receptor atoms, and a Coulomb potential, modeling the polar atomic interactions. As previously observed, the quantitative description of the intermolecular interaction energy by these two terms is rather poor, since there is no correction for the solvent effects, the conformational entropy is not taken into account and the approximation of a rigid receptor ignoring any induced fit effect may be too simple [8]. Although the conformation entropy is actually a difficult quantity to estimate in molecular modeling [9], the solvent effect can be taken in account using higher levels of theory contributing to a better description of the interaction energy [8].

Many of the current models used for modeling the solvent effect (solvation models) are based on a penalty for polar interactions based on the solvent occluded volume after binding [10,11]. Stouten [12] and Luty [13] described an effective empirical solvation model where a distance-dependent Gaussian weight is applied to the solvent occluded volume resulting in an atom ‘occupancy’ parameter. This atom occupancy is then multiplied by an atomic solvation parameter, resulting in the solvation energy for that atom. Those authors also determined the solvation parameters for the atom types typically observed in macromolecules [12]. This method has some advantages for scoring molecular interactions, especially in the context of ligand docking, including the rapid evaluation of the solvent effect in a volume-based approach and the possibility of a pre-computation of the solvation terms in grids speeding-up docking computation [13,14].

A continuum representation of the solvation effect as a dielectric medium can be best described by the Poisson-Boltzmann (PB) model, where meaningful solvation energies can be computed using a high level of theory. However, PB involves a numerical solution of differential equations, requiring an increased computing power [15]. A feasible alternative to PB is the Generalized Born (GB) model, where the Born model for solvation of an ion is extended to molecules of any shape. According to Still approximation [16], the polar contribution to the solvation free energy can be given in GB by:

$$\Delta G_{pol}=-166\left(1-\frac{1}{\varepsilon }\right)\sum_{i=1}^{n}\sum_{j=1}^{n}\frac{q_{i}q_{j}}{f_{GB}}$$ (1)

where *q*_*i*_ and *q*_*j*_ are partial charges, ε is the solvent dielectric constant and *f*_*GB*_ is a function of *r*_*ij*_ and the Born radius [16]. Compared to PB, GB shows an increased efficiency in terms of computation speed and still preserves a linear relation with the former [17].

Despite their efficiency, the rigorous solvation models, such as PB or GB, are still too expensive to be used in the current ligand docking models, where a fast, though accurate, energy evaluation is ought to be achieved for screening a large number of compounds in purchasable screening libraries [18]. Regarding the solvation effect, a compromise has to be established between a rigorous evaluation using a higher theory level and a fast assessment using an empirical model.

Here we evaluated a variation of the Stouten solvation model, as proposed by Verkhivker and coworkers [19] and compared the correlation of solvation energies in this empirical model to experimental solvation energies. This proposed model was found to be in close agreement with experimentally determined solvation energies. Furthermore, we evaluated the model in the context of ligand docking with the software LiBELa [20] and, surprisingly, we found that the introduction of the correction term for solvent effect does not significantly improve the enrichment of binders against decoy molecules. However, when used in prospective screening of small molecule binders, the model was found to correct the overvaluation of the electrostatic term in the binding energy that typically favors highly charged molecules.

## Results

The Stouten-Verkhivker model, or SV model, is based on two very simple assumptions. First, the electrostatic contribution to the solvation energy can be described by a linear function of the square of atomic charges of the molecule, in a classical approach. Thus, an atomic solvation parameter *S* can be defined as

$$S_{i}=\alpha q_{i}^{2}+\beta$$ (2)

where α and β are adjustable parameters and *q*_*i*_ is the charge of atom *i*. Second, the degree of desolvation of a ligand atom by receptor atoms depends on the fragmental volume of the receptor atom and the distance between them, where a Gaussian weight is used for distances:

$$X_{j}=\left(\frac{4\pi }{3}\right)a^{3}\frac{{exp}^{\left[{-r}_{ij}^{2}/(2\sigma^{2})\right]}}{\sigma^{3}}$$ (3)

where *a* is the atomic radius of the atom *j*, *r*_*ij*_ is the distance between atom *i* and atom *j* and σ is a constant. So, similarly to the proposal of Verkhivker [19] and coworkers, the pairwise desolvation energy upon ligand-receptor interaction is given by:

$$E_{ij}^{Solv}=S_{i}X_{j}+S_{j}X_{i}$$ (4)

where the first term in the summation accounts for the desolvation of a ligand atom *i* upon binding a receptor atom *j* and the second term accounts for the desolvation of receptor atom *j*. By summing the contribution of each receptor atom to the desolvation of each ligand atom, the ligand molecular solvation energy is computed. The same holds true for the receptor desolvation.

The proposed model has at least three interesting properties in the context of ligand docking calculations: *i)* first, it is simple and depends on just a few parameters, already used in typical docking calculation, i.e., atomic charge and atomic radius, and does not require the parametrization of new force field parameters; *ii)* it is suitable for calculations in grids [13], which is typical in ligand docking calculations [14], speeding up the actual computation of optimal ligand poses; *iii)* although simple, the model still preserves a dependence with the square of the atomic charge, similar to what is observed in the Born model for hydration of an ion. So, given these properties of the SV model, we then decide to evaluate the model in the context of ligand docking and check if enrichment is improved in typical benchmarks.

### Solvation energies

In the context of ligand docking, any proposed solvation model should have a balance between accuracy, i.e., the ability to compute desolvation energies correlated with experimental solvation energies, and computational efficiency. In an attempt to test the ability of the proposed SV model to reproduce solvation energies, we used the dataset FreeSolv [21], which is a curated database of solvation free energies for small molecules. Since the SV model is pairwise model accounting for the desolvation energy due to the interaction between a ligand and a receptor, we computed the solvation term (*S* in Eqs 2 and 4), assuming that it should be proportional to the solvation energy of the ligand. For this comparison, the atomic charges of the molecules available in the FreeSolv database were recomputed using three different charge models, namely AM1, as available in the program ANTECHAMBER [22], MMF94 and Gasteiger-Marsili model, as available in the program OpenBabel [23].

**Fig 1:**
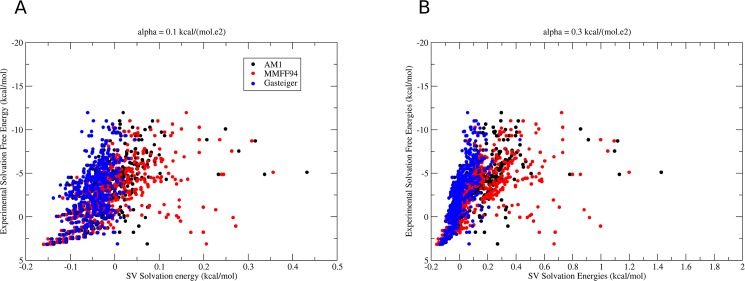
Correlation between experimental and computed solvation energies. Experimentally determined solvation energies for 504 organic compounds and available in the FreeSolv database (vertical axis) and solvation energies computed using the term S in our implementation of the SV model are compared. (A) comparison using α = 0.1 kcal.mol^-1^.*e*^-2^ and (B) α = 0.3 kcal.mol^-1^.*e*^-2^.

The solvation energies as computed using a linear dependency with the square of the atomic charge showed a good correlation with the experimentally determined solvation free energies, as shown in Fig 1 and Table 1. From the three atomic charge models evaluated here, the Gaisteger charge model resulted in the strongest correlation with *r* = 0.71 when α was set to 0.3 kcal.mol^-1^.*e*^-2^). For MMFF94 and correlation coefficient *r* = 0.55 was observed (α = 0.3 kcal.mol^-1^.*e*^-2^) and, for the AM1 charge model, originally used in the FreeSolv database, an *r* = 0.65 was observed under the same conditions.

**Table 1:** Correlation coefficients computed between empirical SV solvation energies and experimental solvation free energies available in the FreeSolv database. N = 504.

Charge Model	α = 0.1 kcal.mol^-1^.e^-2^	α = 0.2 kcal.mol^-1^.e^-2^	α = 0.3 kcal.mol^-1^.e^-2^	α = 0.4 kcal.mol^-1^.e^-2^	α = 0.5 kcal.mol^-1^.e^-2^
AM1	0.61	0.64	0.65	0.65	0.65
MMFF94	0.52	0.55	0.55	0.56	0.56
Gasteiger	0.50	0.66	0.71	0.72	0.72

Decreasing the coefficient α results in slightly decrease in the correlation with experimental solvation energies, as shown in Table 1, and setting it above 0.3 kcal.mol^-1^.*e*^-2^ does not result in improved correlation, suggesting that its optimal value is found around 0.3 kcal.mol^-1^.*e*^-2^.It is remarkable that a simple and fast model can result in a linear response for solvation energies when compared to experimental energies. The correlation between the computed energies is good enough for a solvation model in the context of molecular docking.

### Scoring efficiency

The encouraging correlation between desolvation energies computed with SV model and experimental data prompted us to test this model in retrospective docking simulations. Here, we used our software, LiBELa [20], to dock ligands and decoys against 37 targets of the DUD dataset, 35 targets of the CM-DUD dataset and 12 targets of the DUDE dataset. The enrichments for each target of these benchmarks are summarized in Table 2, Table 3 and Table 4, respectively.

**Table 2:** Enrichment for DUD database showed as percentage of area under curve to linear and semi-logarithmic scales (AUC and logAUC).

Target	Ligands	Decoys	DOCK 6		LiBELa		LiBELa		LiBELa	
			Grid Score		Force Field		Force Field + SV (α = 0.1)		Force Field + SV (α = 0.3)	
			logAUC	AUC	logAUC	AUC	logAUC	AUC	logAUC	AUC
**ACE**	49	1797	2.4	42.8	1.2	51.3	0.8	48.2	3.1	51.1
**ACHE**	107	3892	12.5	69.1	2.9	56.0	2.7	55.9	-1.7	44.9
**ADA**	39	927	30.5	80.5	22.9	80.4	22.4	81.6	21.0	77.9
**AMPC**	21	786	17.6	69.9	29.7	70.6	9.0	46.5	3.4	31.7
**AR**	79	2854	-4.3	27.0	21.1	73.3	19.0	71.2	13.8	66.1
**CDK2**	72	2074	27.0	77.1	29.2	85.5	23.5	80.3	16.2	72.0
**COMT**	11	468	57.7	98.0	20.4	79.9	22.7	68.6	16.8	69.2
**COX1**	25	911	-2.9	44.3	5.7	52.8	8.5	57.0	22.5	62.2
**COX2**	426	13289	-3.3	29.8	24.7	76.4	7.3	62.2	-4.8	36.9
**EGFR**	475	15996	13.1	44.0	26.6	73.8	26.0	75.8	25.6	77.4
**ER AGONIST**	67	2570	16.9	53.1	12.3	68.6	10.7	67.4	6.1	56.9
**ER ANTAG**	39	1448	19.2	72.7	19.1	83.9	19.0	81.6	16.2	80.7
**FGFR1**	120	4550	42.2	77.0	40.9	85.9	39.0	84.8	37.0	81.2
**FXA**	146	5745	26.7	79.9	31.6	89.9	31.8	90.7	35.1	91.7
**GPB**	52	2140	54.2	90.2	3.1	56.4	4.0	57.2	-0.8	45.5
**GR**	78	2947	-6.5	9.2	10.6	59.6	11.6	59.3	12.4	58.8
**HIVPR**	62	2038	-6.1	21.9	5.9	51.1	-0.9	37.8	-4.9	30.3
**HIVRT**	43	1519	19.7	61.3	7.3	54.8	3.1	51.7	-1.4	46.1
**HMGA**	35	1480	-5.1	11.0	35.3	85.2	36.9	85.5	31.1	83.7
**HSP90**	37	979	-5.4	30.7	1.8	56.3	1.5	55.0	3.1	61.4
**INHA**	86	3266	2.3	48.4	4.8	47.3	5.7	51.5	6.1	54.2
**MR**	15	636	-5.1	18.3	34.2	74.5	36.7	79.0	33.2	78.1
**NA**	49	1874	36.9	87.1	31.8	89.7	30.6	90.1	39.3	94.2
**P38**	454	9141	15.2	51.1	19.3	70.5	17.1	67.0	17.5	68.4
**PARP**	35	1351	21.0	77.7	17.0	80.2	10.4	72.5	5.4	63.5
**PDE5**	88	1978	2.9	39.3	13.9	75.6	11.5	71.5	6.7	58.3
**PDGFRB**	170	5980	4.9	38.8	0.5	48.3	1.8	49.8	9.2	55.6
**PNP**	50	1036	18.5	60.9	6.1	51.5	4.1	50.1	2.4	47.0
**PPARγ**	105	3127	22.6	74.4	-1.6	49.7	-0.5	46.5	0.6	51.2
**PR**	27	1041	-5.2	22.9	3.2	51.4	4.0	52.7	4.4	50.9
**RXRα**	20	750	34.2	63.7	11.4	60.9	8.9	61.5	22.0	73.0
**SAHH**	33	1346	26.0	84.6	28.8	79.4	27.5	78.9	22.0	78.1
**SRC**	159	6319	27.7	62.4	33.2	82.6	33.1	80.4	31.5	80.0
**THOMBIN**	72	2456	34.9	81.5	46.9	95.6	38.3	91.4	34.8	89.1
**TK**	22	891	44.1	86.4	16.3	70.9	8.5	67.1	1.9	54.4
**TRYPSIN**	49	1664	34.8	73.8	26.8	84.8	21.4	71.8	13.8	57.0
**VEGFR2**	88	2906	20.8	60.0	29.8	79.0	29.0	74.4	21.0	69.4
**Average**	**32.6 decoys/ligand**		**17.4**	**57.3**	**18.2**	**69.8**	**15.9**	**66.9**	**14.1**	**63.5**
**Median**			**18.5**	**61.3**	**19.1**	**73.3**	**11.5**	**67.4**	**13.8**	**62.2**

**Table 3:** Enrichment for CM-DUD database showed as percentage of area under curve to linear and semi-logarithmic scales (AUC and logAUC). Values of α are given in units of kcal.mol^-1^.e^-2^.

			DOCK 6		LiBELa		LiBELa		LiBELa	
Target	Ligands	Decoys	Grid Score		Force Field		Force Field + SV (α = 0.1)		Force Field + SV (α = 0.3)	
			logAUC	AUC	logAUC	AUC	logAUC	AUC	logAUC	AUC
**ACE**	49	1716	15.4	73.9	0.0	45.0	-1.0	43.5	-0.1	47.6
**ACHE**	108	3781	-0.8	50.2	0.3	50.7	-0.1	52.1	-2.0	43.7
**ADA**	37	1296	2.2	52.7	-2.3	47.7	-2.6	46.3	-2.2	49.6
**AMPC**	21	736	-1.9	48.7	2.0	55.3	0.0	51.2	-5.1	36.3
**CDK2**	57	1996	-5.9	39.2	0.6	55.7	0.4	50.5	-0.1	48.5
**COMT**	12	421	10.8	69.7	7.6	60.1	6.9	59.3	8.5	57.9
**COX1**	24	841	0.8	56.4	15.6	62.2	13.6	59.5	10.1	55.6
**COX2**	420	14666	-5.8	36.2	10.4	68.2	7.2	64.1	2.0	57.9
**EGFR**	573	20021	-0.4	36.2	4.5	55.9	5.3	56.2	4.6	55.2
**ER_AGONIST**	67	2346			23.5	76.2	29.0	82.9	28.7	82.6
**ER_ANTAG**	53	1856			5.3	50.1	-0.2	44.2	-1.6	45.1
**FGFR1**	172	5821	-1.0	46.3	2.1	52.9	3.0	53.4	3.3	52.3
**FXA**	148	5181	3.2	59.0	8.9	72.2	8.4	70.8	7.1	68.8
**GPB**	52	1821	6.4	67.2	-1.2	52.2	0.2	53.3	-1.9	51.5
**GR**	78	2731	-4.6	34.0	-0.2	45.0	1.4	46.2	1.6	46.5
**HIVPR**	62	2171	-0.9	50.0	-3.7	40.9	-0.9	46.6	-2.9	41.5
**HIVRT**	42	1389	-4.0	37.7	3.4	56.0	6.1	59.4	2.3	55.8
**HMGA**	35	1226	-1.7	42.4	8.1	60.0	3.4	59.0	2.8	61.8
**HSP90**	23	806	-8.6	21.2	2.8	55.0	0.7	53.3	3.9	53.3
**INHA**	87	3046	-1.2	50.8	1.3	53.1	2.5	53.6	-0.5	49.2
**MR**	15	526	-1.5	45.9	36.4	73.4	37.7	76.8	28.0	75.1
**NA**	49	1716	14.9	83.8	18.9	86.2	18.4	84.1	17.2	84.3
**PARP**	34	1191	-2.9	44.2	3.1	61.1	2.7	61.3	3.2	59.8
**PDE5**	61	2136	-5.2	34.5	1.0	56.6	-2.2	46.8	0.8	50.6
**PDGFRB**	191	1322			-1.0	51.2	0.0	54.4	-0.8	50.6
**PNP**	26	911	-4.1	36.2	4.1	58.3	4.0	58.4	3.3	57.8
**PPARγ**	86	3011	3.5	59.9	-3.3	42.4	-3.4	42.7	-3.3	42.6
**PR**	27	946	-7.7	29.9	2.6	47.2	3.2	45.9	1.8	44.2
**RXRα**	20	701	12.3	76.6	16.5	55.5	16.5	59.9	10.9	50.9
**SAHH**	40	1401	-3.3	41.4	9.4	66.5	8.3	65.6	7.9	64.3
**SRC**	201	7036	-3.2	42.6	1.5	49.4	1.4	48.5	0.6	46.0
**THOMBIN**	71	2486	1.3	54.8	13.8	73.6	11.4	74.6	14.1	77.0
**TK**	22	771	6.8	68.0	16.1	76.3	16.7	77.9	16.1	74.2
**TRYPSIN**	50	1751	23.0	83.0	14.2	75.3	6.0	60.4	7.6	64.2
**VEGFR2**	87	330	7.4	58.9	1.7	56.1	0.2	54.2	-1.9	48.3
**Average**					**6.4**	**58.4**	**5.8**	**57.6**	**4.7**	**55.7**
**Median**					**3.1**	**55.9**	**3.0**	**54.4**	**2.3**	**52.3**

**Table 4:** Enrichment for DUDE database showed as percentage of area under curve to linear and semi-logarithmic scales (AUC and logAUC).

Target	Lig	Dec	DOCK 6		LiBELa		LiBELa (α = 0.1)		LiBELa (α = 0.3)		
			Grid Score		Force Field		Force Field+SV		Force Field + SV		
			logAUC	AUC	logAUC	AUC	logAUC	AUC	logAUC		AUC
**CP2C9**	183	7574	-3.9	30.1	-0.1	48.4	-1.5	46.0		-1.4	45.5
**CXCR4**	122	3414	-5.3	35.7	-1.7	43.8	-1.3	47.8		-3.1	44.7
**GRIK1**	152	6617	10.0	58.8	10.2	64.8	11.0	67.3		11.8	68.4
**MK10**	186	6714	-3.1	39.7	2.0	55.6	1.1	56.0		-0.3	52.6
**XIAP**	129	5213	9.9	58.5	5.9	61.4	8.0	66.8		6.5	65.6
**MCR**	193	5240	-11.0	13.7	4.8	55.3	5.3	56.2		4.6	55.2
**THB**	167	7641	5.18	45.3	10.5	67.2	11.4	67.1		9.2	64.6
**HIVINT**	211	6756	6.9	51.4	4.3	59.0	5.9	63.7		4.9	63.4
**KITH**	132	2866	-7.7	30.4	6.5	63.5	9.6	70.4		9.3	71.7
**PUR2**	201	2725	30.8	81.5	28.0	85.9	32.5	88.6		31.1	87.0
**LKHA4**	244	9477	17.1	67.3	6.7	61.1	6.8	61.3		4.2	58.3
**PPARD**	288	13232	10.7	63.0	3.7	57.9	5.9	61.1		7.1	62.4
**DYR**	566	17384	16.1	51.6	13.6	61.8	13.1	63.9		7.8	58.2
**Average**			**5.8**	**48.2**	**7.3**	**60.4**	**8.3**	**62.8**	**7.0**		**61.3**
**Median**			**6.9**	**51.4**	**5.9**	**61.1**	**6.8**	**63.7**	**6.5**		**62.4**

**Fig 2:**
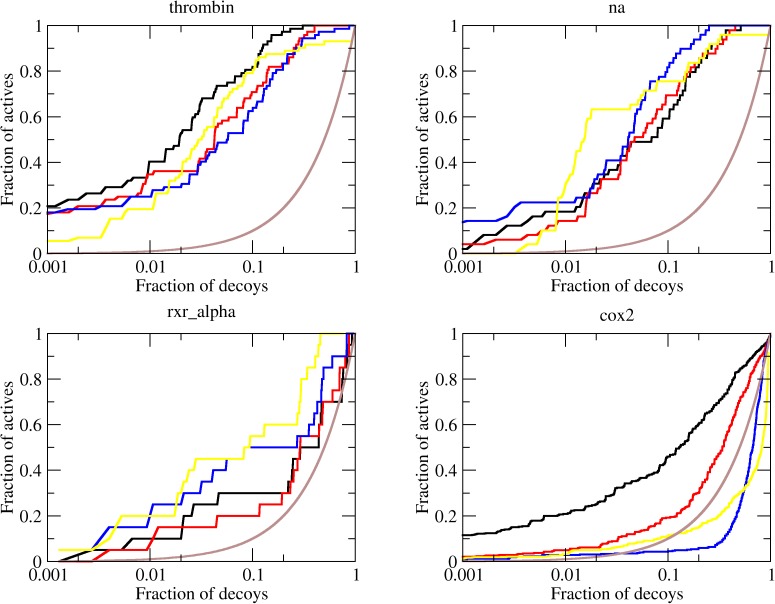
ROC curves for representative DUD targets. The brown line shows the enrichment expected to for a random distribution of ligands and decoys. The enrichments obtained with FF (black line), SV (α = 0.1 kcal.mol^-1^.*e*^-2^, red), SV (α = 0.3 kcal.mol^-1^.*e*^-2^, blue) and DOCK6 (yellow) are shown. The complete set of ROC curves for DUD targets is available in S1 File.

First of all, the well-established and validated tool DOCK6 was used as a positive control in the enrichment calculations. The DUD data set was submitted for docking calculations using automatically prepared receptor files and a pure force field scoring function (grid score). The entire calculation took about 336 minutes per target, averaging over the entire dataset, and the enrichment was then calculated using bash and python scripts. As shown in Table 2, an average logAUC of 17.4 was achieved with an average AUC of 57.3% (median 18.5 and 61.3%, respectively), which is in agreement with the enrichments reported by the DOCK6 developers [24]. For four targets, ADA, COMT, GPB and TK, DOCK6 achieves notable enrichments, especially considering the first decade of the semi-log graph, reaching logAUC values of 30.5, 57.7, 54.2 and 44.1 respectively (Fig 2). Interestingly, TK was previously shown to be a difficult target to obtain a good enrichment against its own decoys [25] when tested using DOCK 3.5.54, indicating that the interaction model used by DOCK6 is indeed accurate.

The same ligands and decoys of the DUD dataset were also used for docking simulations in LiBELa, using a pure AMBER force field scoring function, similar to the grid score implemented in DOCK6 [20]. The docking calculations took about 175 minutes per target set on average and resulted in an average logAUC of 18.2 with an average AUC of 69.8% (median 19.1 and 73.3%, respectively). The results obtained with the hybrid approach as implemented in LiBELa were shown to be efficient using a pure force field scoring function and are in agreement with the results previously reported for this interaction model [20].

Considering that the solvent has important effects in ligand binding, one would expect that an appropriate treatment of these effects would lead to even better results in terms of enrichment of actual ligands against decoys. We then used the SV solvation model in docking simulations with the DUD dataset. For these calculations, the parameter α was varied between 0.05 and 0.4 kcal.mol^-1^.*e*^-2^ and the parameter β was kept fixed at -0.005 kcal.mol^-1^. Surprisingly, a worse average enrichment was achieved using the SV model when compared to a pure force field scoring function, as shown in Table 2, Fig 2 and S1 File. Averaging over 37 DUD targets, the FF+SV model achieved a logAUC of 15.9 with an AUC of 66.9% (median 11.5 and 67.4%, respectively), when the parameter α was set to 0.1 kcal.mol^-1^.*e*^-2^, i.e., the average enrichment was 13% inferior to the results achieved using a pure FF model, while the median decreased by 40%. An increase in the value of α resulted in a decrease in the average enrichment observed for this dataset, with an average logAUC of 14.1 (median in 13.8) and average AUC of 63.5 (median 62.2) for α = 0.3 kcal.mol^-1^.e^-2^ (Table 2). Different configurations of the solvation model with values of α ranging from 0.05 to 0.4 kcal.mol^-1^.e^-2^ were also tested resulting in decreased enrichments in all of these scenarios (S4 File).

As compared to the enrichment obtained in the pure FF model, considerable differences were observed for COX2, AMPC, TK, TRYPSIN and CDK2, all targets with highly polar pockets. In these cases, the pure FF model resulted in better enrichments than the FF+SV interaction model. Curiously, COX1 was one example where the introduction of solvation effect in the energy model improved the enrichment. Similar to COX2, COX1 has a partially buried polar (charged) binding site that recognizes small and charged molecules. The difference in the results observed for COX1 and COX2 may suggest that minor issues related to the ligands/decoys choice, for example, could play a role here.

**Fig 3:**
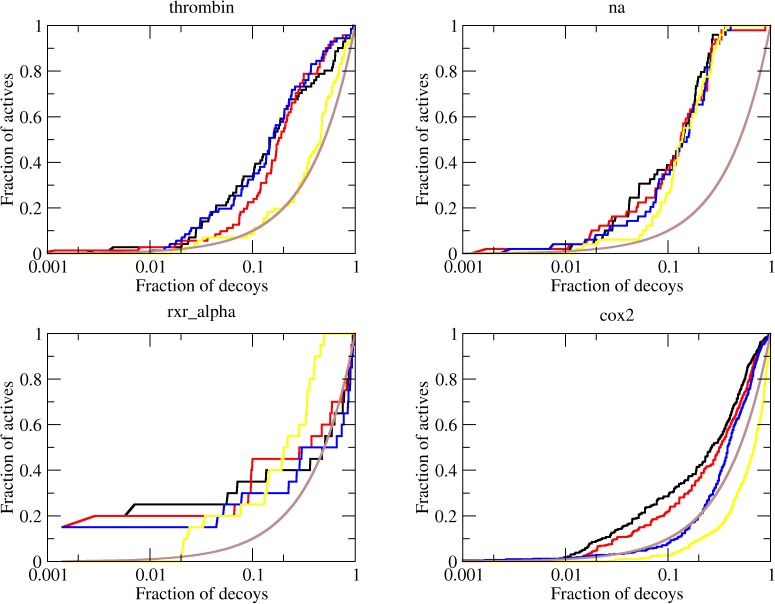
ROC curves for representative CM-DUD targets. The brown line shows the enrichment expected to for a random distribution of ligands and decoys. The enrichments obtained with FF (black line), SV (α = 0.1 kcal.mol^-1^.*e*^-2^, red) and SV(α = 0.3 kcal.mol^-1^.*e*^-2^, blue) are shown. The complete set of ROC curves for CM-DUD targets is S2 File.

The original DUD dataset was further shown to be imprecise in the distribution of charged molecules among ligands and decoys [11], with an increased fraction of charged ligands as compared to decoys. This inaccuracy could make ligands artificially more attractive to polar binding sites. The developers then released a charged-matched DUD (CM-DUD), where this inaccuracy was fixed. We also used the CM-DUD for docking simulations to assess whether the polar solvation model was artificially penalizing the actual ligands more than the decoys due their increased tendency to be charged. The enrichments observed for individual targets are shown in Table 3, Fig 3 and S2 File.

For the CM-DUD, an average logAUC of 6.4 with an AUC of 58.4% was observed for the pure FF energy model implemented in LiBELa, much smaller than the average logAUC observed for the original DUD but still comparable with the logAUC observed for DOCK6 grid score (2.7 with an average AUC of 52.0%), as shown in Table 3 and Fig 3. The introduction of the solvation model into the scoring function resulted again in a decrease in the average logAUC to 5.8, for α = 0.1 kcal.mol^-1^.*e*^-2^ and 4.7 for α = 0.3 kcal.mol^-1^.*e*^-2^.

These results underline important issues in terms of scoring functions for docking. First, the volume-based SV-model does not result in significant improvement in the enrichments of most targets when compared to a pure force field scoring function, despite its good correlation with experimental solvation energies. In original DUD or CM-DUD, a decrease of around 20–25% in the average enrichment is observed upon the introduction of the solvation effect as modeled by the SV model with α set to 0.3 kcal.mol^-1^.*e*^-2^. Second, the Coulomb model for electrostatic interactions is probably a weak model for quantifying macromolecular interactions. We come to this conclusion by comparing the performance of LiBELa and DOCK6 with the results previously reported for DOCK3.7, where a high enrichment was achieved for the CM-DUD dataset. That tool uses DELPHI [26], which relies on the numeric solution of the Poisson-Boltzmann for the evaluation of polar interactions [11]. Considering that the charge-pairing of ligands and decoys are the most important change between DUD and CM-DUD, it is plausible to think that the stronger electrostatic model was an important reason for the increased enrichment observed for DOCK3.7.

**Fig 4:**
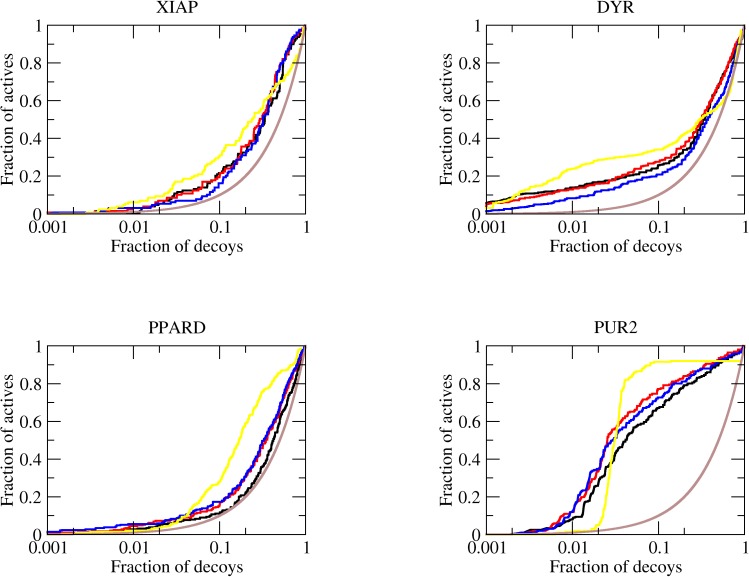
ROC curves for representative DUDE targets. The brown line shows the enrichment expected to for a random distribution of ligands and decoys. The enrichments obtained with FF (black line), SV (α = 0.1 kcal.mol^-1^.*e*^-2^, red), SV(α = 0.3 kcal.mol^-1^.*e*^-2^, blue) and DOCK6 (yellow) are shown. The complete set of ROC curves for DUDE targets is available in S3 File.

Finally, we decided to assess whether sampling effects could be playing a role in the average results observed for DUD and CM-DUD. Despite the good decoy-to-ligand ration (around 33 decoys per ligand), these data sets have just a few ligands in some cases. We then decided to use twelve targets of the DUD-Enhanced (DUDE) dataset, created from an increased number of actives molecules and keeping the decoy-to-ligand ratio around 33. For DUDE, interesting results were observed: for the pure FF model, an average logAUC of 7.3 was observed with and AUC of 60.4% (median 5.9 and 61.1%), as shown in Table 4, Fig 4 and S3 File. The introduction of the SV correction term to account for solvent effects resulted in a slight increase in the enrichment, resulting in an average logAUC of 8.3 and an AUC of 62.8% when α was set to 0.1 kcal.mol^-1^.*e*^-2^ (Fig 4). Setting the parameter α above this threshold resulted in a systematic decrease in the average logAUC, revealing an optimal value between 0.1 and 0.2 kcal.mol^-1^.*e*^-2^. Two interesting features can readily be observed in this result. First, there is an overall improvement of enrichment, i.e., there are small improvements for 10 out of the 13 target targets when α was set to 0.1 kcal.mol^-1^.*e*^-2^. This can be observed by the increase in the median logAUC together with the increase of the average logAUC. This observation suggests that the SV model result in consistent improvement in this larger dataset. Second, the targets with the higher improvement, PUR2, KITH, PPARD and GRIK1 have in common buried or partially buried active sites and polar interactions with the ligand though charged residues. For active sites with these features, a correction for the solvent effects is expected to play a significant role, resulting in improvement of the enrichment.

Are the errors introduced in the scoring function due to sampling issues or confined to the scoring of molecular interactions? This is not a trivial question to address. In order to tackle this issue, we run docking simulations (self-docking) for 1,031 ligands of the SB2012 dataset using three different energy models, namely a pure FF model and FF+SV with α parameter set to 0.1 and 0.3 kcal.mol^-1^.e^-2^. The results are summarized in the Table 5. These results show that the introduction of a correction term to take into account the solvent effects does not result in significant increase of the root mean square deviation or of the rate of success at lower values of the parameter α, i.e., up to 0.2 kcal.mol^-1^.*e*^-2^. Increasing this parameter gradually increases the deviation, although keeping very low RMSD values as compared to DOCK6, as one would expect for a hybrid docking such as the used in LiBELa. Taken together the results shown in Table 5 indicate that sampling issues are not expected to play a relevant role in the decreased enrichment observed.

**Table 5:** Statistics of pose reproduction of the SB2012 dataset using DOCK6 and LiBELA using a pure FF energy model and FF+SV energy model.

	DOCK6	LiBELA FF	LiBELA FF+SV α = 0.1 kcal.mol^-1^.e^-2^	LiBELA FF+SV α = 0.3 kcal.mol^-1^.e^-2^
**Average RMSD (Å)**	5.91	0.86	0.90	1.34
**Median RMSD (Å)**	5.29	0.50	0.48	0.49
**RMSD < 1.0 Å**	17%	86%	86%	82%
**RMSD < 2.5 Å**	32%	95%	95%	90%
**RMSD < 3.0 Å**	40%	96%	95%	90%

## Discussion

Here we evaluated a variation of the widespread Stouten solvation model in the context of ligand docking. Despite the good correlation of the solvation energies computed by the SV model and experimentally determined solvation energies, the SV model resulted in decreased enrichments when used in retrospective docking simulation of gold standard benchmarks such as DUD and CM-DUD. This apparent paradox raises important questions. First, is the model inconsistent? And is the solvation treatment useful in the context of molecular docking?

The first question is somewhat tricky. How can a solvation model be correlated to experimental data and still lead to worse results? Looking at other empirical solvation models already proposed, we found similar results. For example, Mysinger and Shoichet observed that 20 out of the 40 DUD targets had better enrichments when no desolvation penalties were computed than when their solvent-excluded volume model (SEV) model was applied [11]. The average logAUC for no desolvation and for the SEV model were 14.3 and 15.0 with average AUC values of 68.8 and 68.7%, respectively, indicating again that the introduction of an empirical solvation function into their docking scoring function resulted in minimal improvement of the enrichment results, if any. Worth of note, TK and TRYPSIN were two targets with better enrichments using no desolvation, similarly to what was observed in this work with the SV model. The results shown for our SV model and for SEV suggest that empirical models for solvation treatment are still in their infancy and may represent an open road for future developments, necessary for the achievement of a more precise model for molecular interactions in the context of ligand docking.

Additionally, Coleman and coworkers revised the results obtained in the SAMPL4 challenge for solvation energies computed for 47 compounds using AMSOL, according to the default ZINC processing pipeline [27] and used by DOCK3.7. They found that the solvation energies as computed with this protocol were the worst predictions submitted to the challenge [28]. This conclusion highlights the current need for better fast empirical models applicable in the docking context and also provides an explanation for the lack of actual improvement upon the introduction of the solvation penalty in retrospective tests with DOCK. It should be mentioned that, in this context, our SV model showed a good correlation with experimental data, even when simple and empirical atomic charges were used for parametrization of the molecules.

**Fig 5:**
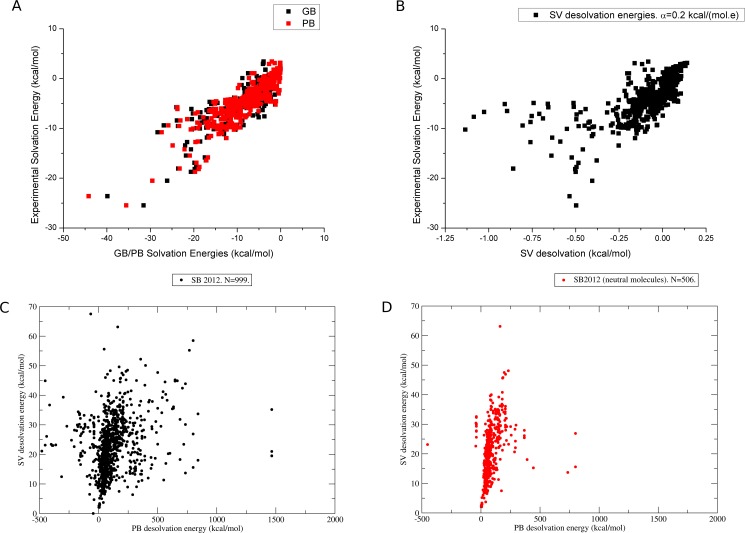
Correlation between experimental solvation data and solvation energies from multiple models. (A) Correlation between experimental solvation data for the FreeSolv dataset (N = 643) and solvation energies computed with GB (black squares) and PB (red squares). The Pearson correlation coefficient is *r* = 0.86, Spearman, *s* = 0.87 and Kendall coefficient is *k* = 0.70 for GB and *r* = 0.88, *s* = 0.98 and *k* = 0.72 for PB. (B) Correlation between experimental solvation energies for the FreeSolv dataset and SV computed solvation energies. *r* = 0.67, *s* = 0.75 and *k* = 0.57. (C) Correlation between desolvation energies for 999 receptor-ligand complexes of the SB2012 dataset computed with PB (horizontal axis) and SV (vertical axis). *r* = 0.23, *s* = 0.40 and *k* = 0.29. (D) Correlation between desolvation energies for complexes of the SB2012 with neutral ligands (N = 506). *r* = 0.21, *s* = 0.57 and *k* = 0.43.

One important source of errors in our model may be related to the evaluation of the electrostatic term in the binding energy. As already pointed out, the effect of long range interactions is best taken into account by continuum electrostatic models, where the dielectric constant and ionic strength can very through space [29]. The approach used to evaluate the polar interactions used in LiBELa is certainly limited by assuming a homogeneous and isotropic media. This is in part compensated by assuming a distance-dependent dielectric ‘constant’, i.e., ε = *r*_ij_, but certainly results in accumulation of errors. The same analysis holds true for the solvation energies, which are primarily based on a penalty for polar interactions with a short- to medium range limit given by the Gaussian envelope function used. The consequences of these limitations can be observed in the performance of the SV model when compared to continuum methods such as GB and PB for a set of complexes of the SB2012 dataset. Continuum models such as GB and PB are very effective in reproducing the overall profile of solvation energies. Calculations done to the molecules of the FreeSolv database show a linear correlation of experimental solvation with energies computed with these continuum models (Fig 5A). The same comparison between experimental solvation energies and SV computed energies (Figs 1 and 5B) still shows that SV can predict the overall solvation profile for the organic molecules in the database but with lower correlation than continuum models, as expected. We also evaluated the calculations in a scenario of desolvation due to binding with the complexes of SB2012 dataset, where experimental desolvation energies are no available, but the empirical SV model can be compared to continuum models. When 999 molecules are compared for their solvation energies (Fig 5C), some correlation can be observed, although for some molecules, a random dispersion is observed. Interestingly, when the dataset is filtered to preserve neutral molecules only (Fig 5D, N = 506) the correlation between solvation energies computed with PB and SV increases substantially, revealing the weakness of the model to handle highly charged molecules. We therefore foresee opened opportunities for the improvement of solvation models by the adoption of more robust overall treatment of the electrostatics for molecular interactions.

Many results have been reported in the literature comparing the efficiency of a simpler interaction model with more robust models such as MM-GBSA or MM-PBSA. For example, Zhang and coworkers recently showed a massive parallel pipeline for virtual screening based on ligand docking with Vina and pose rescoring using the MM-GBSA as implemented in Amber [30]. Using the higher theory level for rescoring, a better enrichment of the DUDE benchmark was reached when compared to the results of docking alone. The authors reported an average AUC of 66.4% (median 68.3%) when using the Vina energy model and 71.1% (median 70.3%) after the rescoring. This 7% in increase of the average area under the curve of ROC plots was obtained at the price of 5 hours of massive parallel computation on 15,000 CPUs, spent only on rescoring. These results suggest that the improvement of the current interaction models used for ligand docking are not just necessary but may also be sufficient to make pointless the rescoring of docking poses using end-point methods.

Graves and coworkers also evaluated the MM-GBSA model for rescoring docking poses generated by DOCK3.5 [8]. From 33 tested ligands, MM-GBSA recovered 23 actual binders that were considered false negatives in docking. On the other hand, 10 true negatives according to the docking model were introduced as false positives after rescoring. This example shows that even a more robust solvation model such as GB can result in important errors during the ranking of a ligand-receptor complex, suggesting the modeling of such interactions can be challenging even for the higher theory levels.

The results briefly reviewed above reveal that even when corrected for solvation energies using robust solvation models, the inaccuracies in the interaction model seem to equate the inaccuracies in the solvation model resulting in results that are marginally better but very expensive in terms of computational time.

The Stouten model could be a choice for a fast solvation model used in molecular interaction modeling. When originally proposed, this fast method was shown effective to reproduce correct conformations of BPTI, comparable with conformations observed in explicit solvent simulations [12]. Variations of this model are already used for molecular docking in the program AutoDock, for example [10]. Evaluating the performance of the variation of the Stouten model proposed by Verkhivker and coworkers, we found that the introduction of the solvent effect into the scoring function does not improve the enrichment of known ligands against decoys on DUD or CM-DUD and results in modest improvement in DUDE targets.

**Fig 6:**
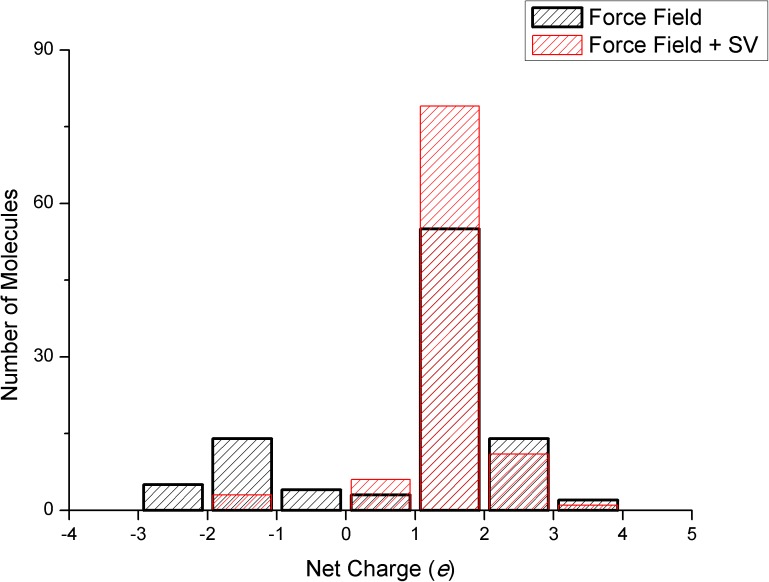
Distribution of net charge for the top scored molecules in VS. Distribution of net charge for the top scored molecules found in a virtual screening campaign using PPARγ as the target. For the SV model, the parameter α was set to 0.2 kcal.mol^-1^.e^-2^.

The question still remains: is the solvation correction useful in the context of molecular docking? It definitely is! We tested the performance of our SV model in a scenario closer to a real application, in a virtual screening campaign using the nuclear receptor PPARγ as the target. This receptor has a buried and bulky active site with three ‘arms’ [31]. One of these arms, is very polar and the other two arms are mainly hydrophobic. The natural ligands of this receptor are fatty acids and several synthetic ligands have already been described in the literature. Worth of note, the thiazolidinediones (TZDs), such as pioglitazone and rosiglitazone, are high affinity ligands of the receptor PPARγ. After screening the dataset ChEMBL Drugstore [32], as available in ZINC [27], we analyzed the net charge of the top one-hundred molecules, ranked by binding energy using a pure FF model and also using FF+SV interaction model. As shown in Fig 6, the introduction of the solvation correction term, reduced the excess electrostatic energy term, reducing the fraction of molecules with charge -2 and -3 among the top scored molecules. Since the receptor is expected to typically bind neutral or -1 charged molecules, the solvent correction is shown to be useful to penalize the polar interactions and avoid the overvaluation of the electrostatic term in the binding energy.

In conclusion, we found that after the adjustment of the parameter alpha, the SV solvation model shows a good correlation with experimental solvation energies. However, the introduction of this model into a docking scoring function does not improve the enrichment of actual binders against decoys. In contrary, the enrichment was reduced about 20%. These results, in line with recent findings for empirical solvation functions, highlight the need for better interaction models that could be useful in the context of molecular docking. In addition, the introduction of the correction term clearly showed an ability to correctly penalize molecules with net charges -2 and -3 *e* making them less attractive in the context of virtual screening [33].

## Experimental

### The Stouten-Verkhivker solvation model

The original Stouten model for solvation free energy is based on the atomic exposal to solvent, i.e., the occupancy of protein atoms around a ligand atom, with a Gaussian envelope function. This occupancy term is then multiplied by an atomic solvation term that depends on the atom type [12]:

$${\Delta G}_{i}^{solv}=\left[\sum_{j}Vol(j)∙{exp}^{\left(\frac{{-r}_{ij}^{2}}{2\sigma^{2}}\right)}\right]∙[SolPar(i)]$$ (5)

Verkhivker and coworkers gave a new view of the intrinsic solvation parameter by proposing that, phenomenologically, each atom has an ‘affinity’ by the solvent that can be given by a function of the square of the atomic charge [19]. Although this proposal was used in a different context, with a softcore potential, a final empirical solvation model can be defined as:

$$E_{ij}^{solv}=(S_{i}f_{j}+S_{j}f_{i}){exp}^{\frac{\left[\frac{{-r}_{ij}^{2}}{2\sigma^{2}}\right]}{\sigma^{3}}}$$ (6)

where *f* is the solvent volume displaced by an atom and *S* is a function of the square of the atomic charge, $S_{i}=\alpha q_{i}^{2}+\beta$. This model, hereinafter Stouten-Verkhivker, or SV model, was evaluated in this work with α=0.2 kcal.mol^-1^.*e*^-2^, β=-0.005 kcal.mol^-1^ and σ=3.5 Å, unless otherwise stated.

## Assessment of the SV model as an additional term in docking scoring function

The test set SB2012 [24] was used to assess the performance of the docking scoring function. This dataset is composed by 1,043 receptor-ligand complexes provided with crystallographic ligands already placed in their receptors binding site, so that the binding energy can be calculated taking the conformational search out of equation. Receptor and ligands were used as provided, i.e., without any additional preparation. For this evaluation, the program DOCK6.7 [24] was employed for the sake of comparison, using the Grid Score. The same receptor-ligand complexes were scored in our algorithm, LiBELa [20], where a pure Amber Force Field energy evaluation is combined with the SV solvation model as described above:

$$E_{LiBELa}=\sum_{i}^{receptor}\sum_{j}^{ligand}\frac{q_{i}q_{j}}{\varepsilon r_{ij}}+\frac{A_{ij}}{r_{ij}^{12}}-\frac{B_{ij}}{r_{ij}^{6}}+S_{i}X_{j}+S_{j}X_{i}$$ (7)

The energy evaluation in pre-computed grids was also employed in LiBELa for both energy models. From the 1,043 receptor-ligand complexes, 1,031 were actually docked using LiBELa and 953 were docked using DOCK6.

### Assessment of the SV model in enrichment tests

The SV solvation model was also evaluated in a typical virtual screening scenario, where a large number of compounds are computationally scrutinized looking for actual binders. This assessment consisted of docking simulations of known ligands and decoys against 37 targets of the directory of useful decoys (DUD) [25]. The Adjusted logAUC (logAUC) was used as a metric for enrichment together with the area under the curve (AUC). Here, the term *enrichment* describes the ability of the algorithm to populate the top of list of docked molecules sorted by docking energies with known binders against decoys. The Adjusted logAUC measures the area under the curve of a semi-log representation of the false positive against true positive rates and subtracts the area expected for a random disposition of ligands and decoys in the ranked results. By using this log scale, the logAUC gives the same weight to the enrichment in the very beginning of the dataset (0.1% to 1% of decoys) to the mid-early enrichment (1–10% of decoys) and to the late enrichment (10–100% of decoys) [11]. Since the distribution of charged and neutral molecules among the decoys in the original DUD dataset was found to be different from the distribution in the ligands, a new version of the DUD was proposed by its developers, the charge-matched DUD or CM-DUD [11]. Soon after, the same developers also proposed the DUD-enhanced, or DUDE [34]. The CM-DUD and a subset of DUDE were also used for the assessment of the SV solvation model in retrospective studies.

The protocol for receptor and ligand preparation was the same for these three benchmarks. The receptors were prepared with UCSF Chimera [35], where missing hydrogen atoms were added, and atom types and charges were attributed according to Amber FF9SB force field [36]. Ligands and decoys were used as provided in SYBYL mol2 files, according to the default ligand preparation of the database [27,37]. In cases where molecules were not distributed in SYBYL mol2 files (e.g., CM-DUD), the 3D structures were generated by BABEL [23] using MMFF94 charge model. When applicable, the program pymdpbsa, part of the AMBER package, was used for estimating solvation energies using GB and/or PB models.

## Docking

Docking of ligands and decoys were performed against their own target using LiBELa [20]. LiBELa (*Ligand Binding Energy Landscape*) is a hybrid algorithm based on the superposition of a search ligand on a reference ligand already placed in the receptor binding site. This superposition is achieved by matching the molecular volume and charge distribution as the target function using the MOLSHACS algorithm [38]. Afterwards, the pose of this initially placed ligand is optimized once again in the Cartesian space (as a rigid body) using the binding energy as the objective function. The ligand flexibility is treated by an *on-the-fly* generation of an ensemble of conformers through a stochastic search of rotatable bonds with the genetic algorithm implemented in the OpenBabel API [23]. Each conformer is overlaid on the reference ligand and the best conformer is then optimized inside the active site as a rigid body in order to generate a final and low-energy binding pose. The augmented Lagrangian [39–41] algorithm was used for optimization of the molecular overlay followed by the optimization of the binding pose using the dividing rectangles algorithm [42]. Both methods were used as implemented in the NLOPT library [43] with a 10^-6^ relative tolerance and a timeout of 30 seconds. The ligands were allowed to translate 12 Å in each direction during the Cartesian search and to perform a full rotation around Euler’s angles. Twenty conformers were generated for each ligand and the best two conformers, as judged by the initial binding energy, were used in the second Cartesian search. The best scored conformer was used for ranking purposes. A cubic grid box with 30 x 30 x 30 Å was used for pre-computation of the potential energies and docking simulations [13]. The grid points were equally spaced by 0.3 Å in all directions. For electrostatic potential energies, the dielectric ‘constant’ was set to the interatomic distance, as previously proposed [29].

Docking simulations using the same benchmarks were also performed with DOCK6.7 using Grid Score with parameters similar to those used in LiBELa, whenever possible. The cluster of spheres generated by SPHGEN within a 10 Å radius from the crystallographic (reference) ligand were selected as shape descriptors. Interaction grids were then computed in a box encompassing the selected spheres with a 5 Å buffer. The grid was computed using a 0.3 Å spacing using Amber FF99 atomic parameters. For docking, a maximal number of 500 orientations was used allowing a chemical matching between receptor and ligand atoms. Ligand flexibility was treated with the anchor-and-grow method with a further minimization of poses in a 100 simplex cycles.

## Supporting information

S1 File ROC curves for all DUD targets. The brown line shows the enrichment expected to for a random distribution of ligands and decoys. The enrichments obtained with FF (black line), SV (α = 0.1 kcal.mol^-1^.*e*^-2^, red), SV (α = 0.3 kcal.mol^-1^.*e*^-2^, blue) and DOCK6 (yellow) are shown.
(DOCX)

S2 File ROC curves for all CM-DUD targets. The brown line shows the enrichment expected to for a random distribution of ligands and decoys. The enrichments obtained with FF (black line), SV (α = 0.1 kcal.mol^-1^.*e*^-2^, red), SV (α = 0.3 kcal.mol^-1^.*e*^-2^, blue) and DOCK6 (yellow) are shown.
(DOCX)

S3 File ROC curves for all DUDE targets. The brown line shows the enrichment expected to for a random distribution of ligands and decoys. The enrichments obtained with FF (black line), SV (α = 0.1 kcal.mol^-1^.*e*^-2^, red), SV(α = 0.3 kcal.mol^-1^.*e*^-2^, blue) and DOCK6 (yellow) are shown.
(DOCX)

S4 File Enrichment data for DUD targets for different sets of α values in SV model. (XLSX)
