# Supplementary material for: Towards a critical evaluation of an empirical and volume-based solvation function for ligand docking
Source: PLoS One. 2017 Mar 21;12(3):e0174336. doi: 10.1371/journal.pone.0174336 (PMC5360343; doi:10.1371/journal.pone.0174336)

**S1 File. ROC curves for all DUD targets**. The brown line shows the enrichment expected to for a random distribution of ligands and decoys. The enrichments obtained with FF (black line), SV (α=0.1 kcal.mol^-1^.e^-2^, red), SV (α=0.3 kcal.mol^-1^.e^-2^, blue) and DOCK6 (yellow) are shown.


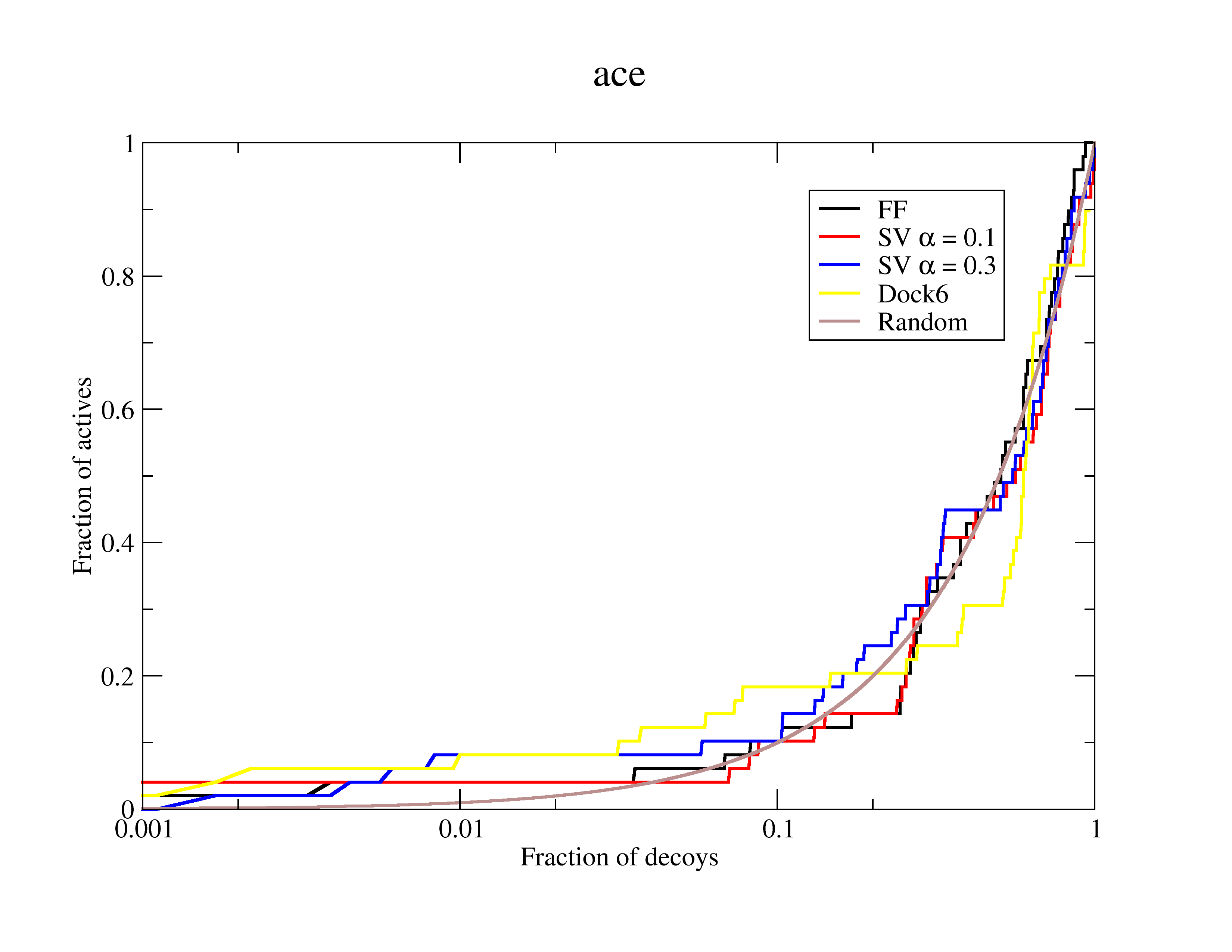

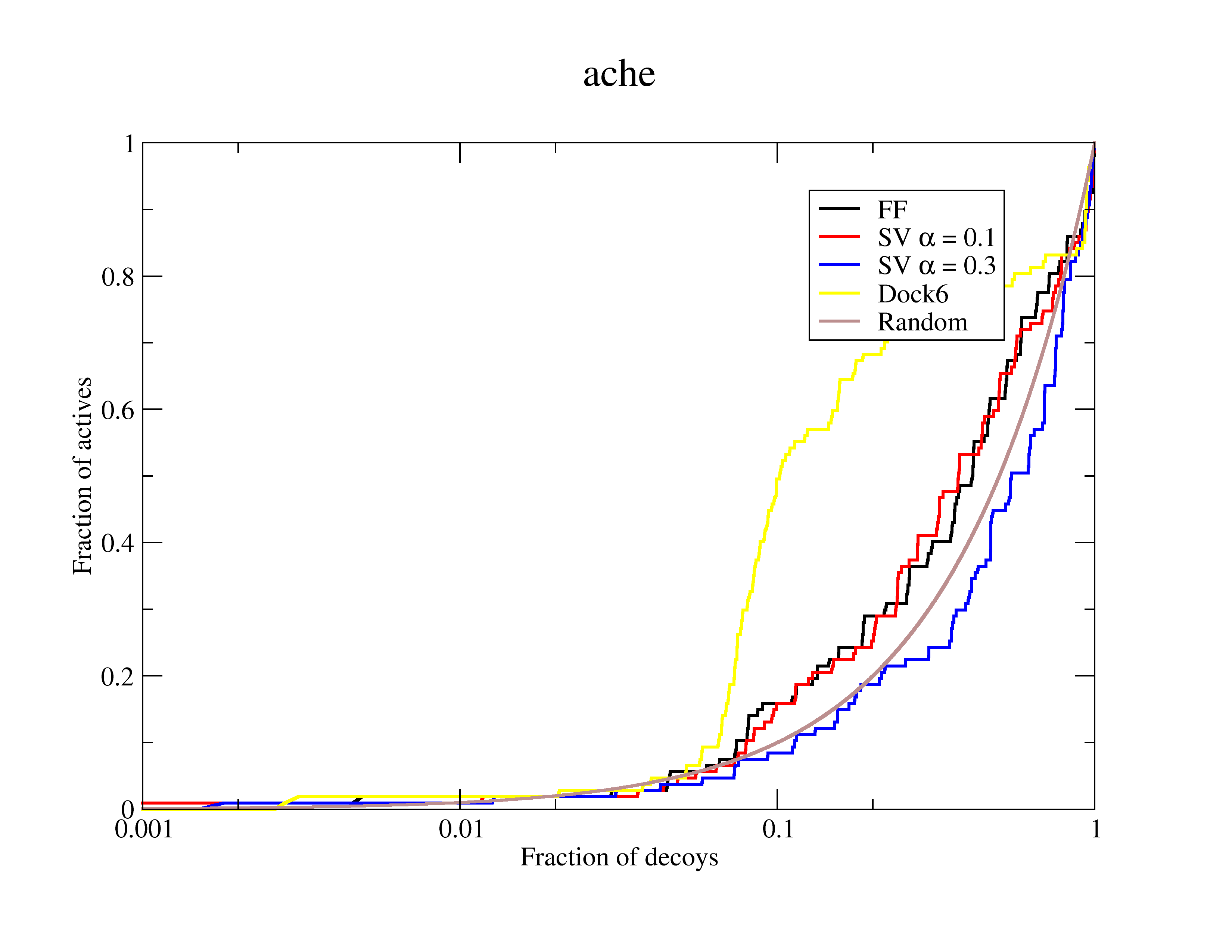

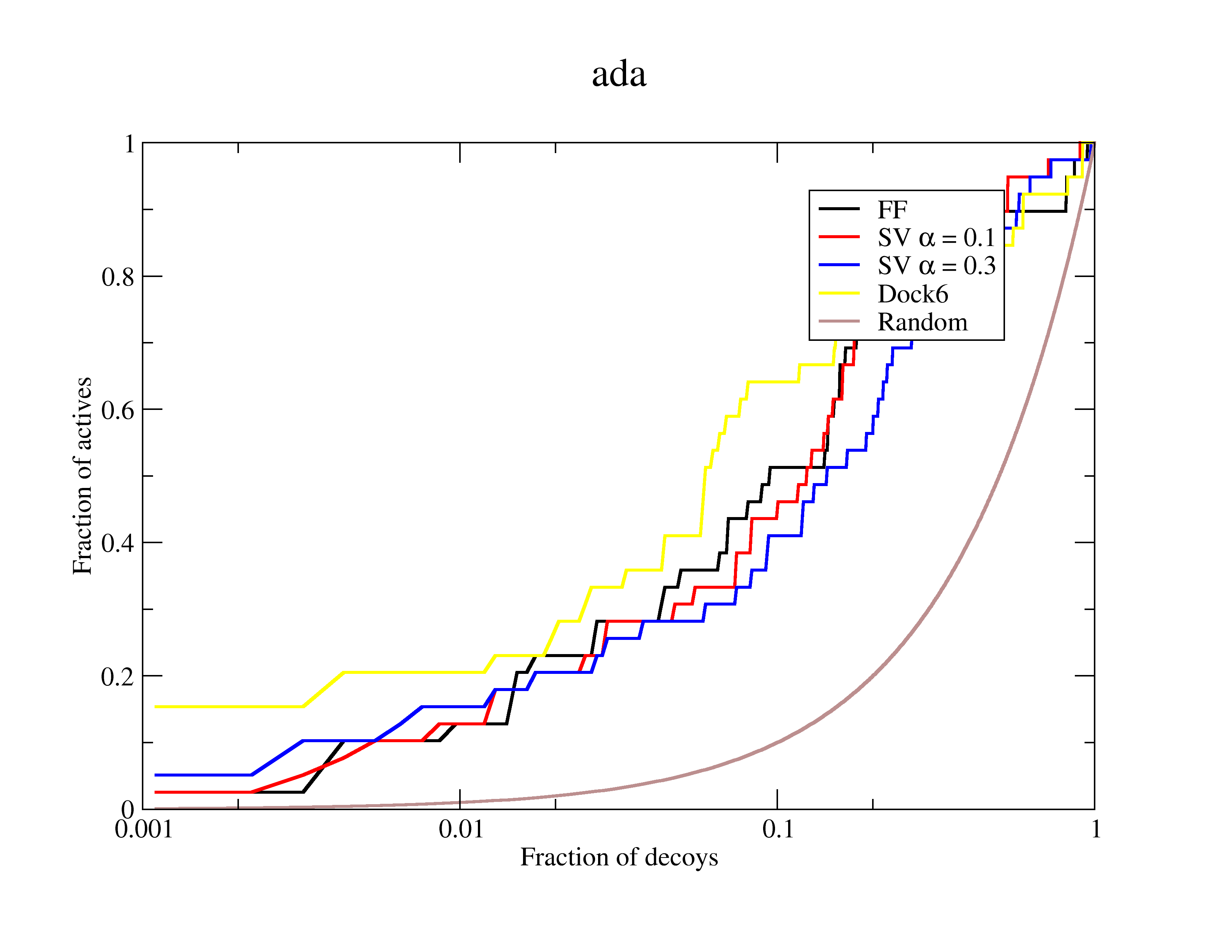

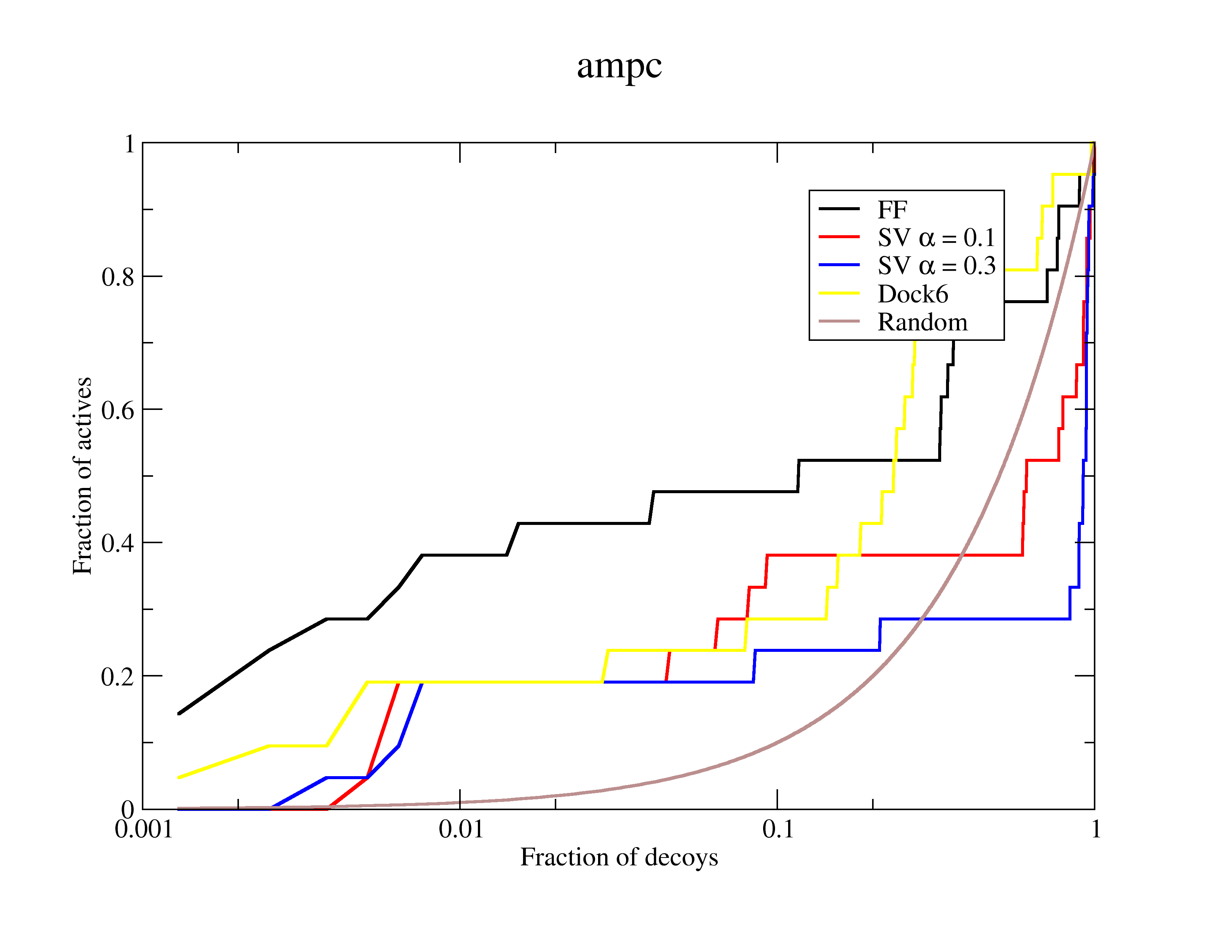

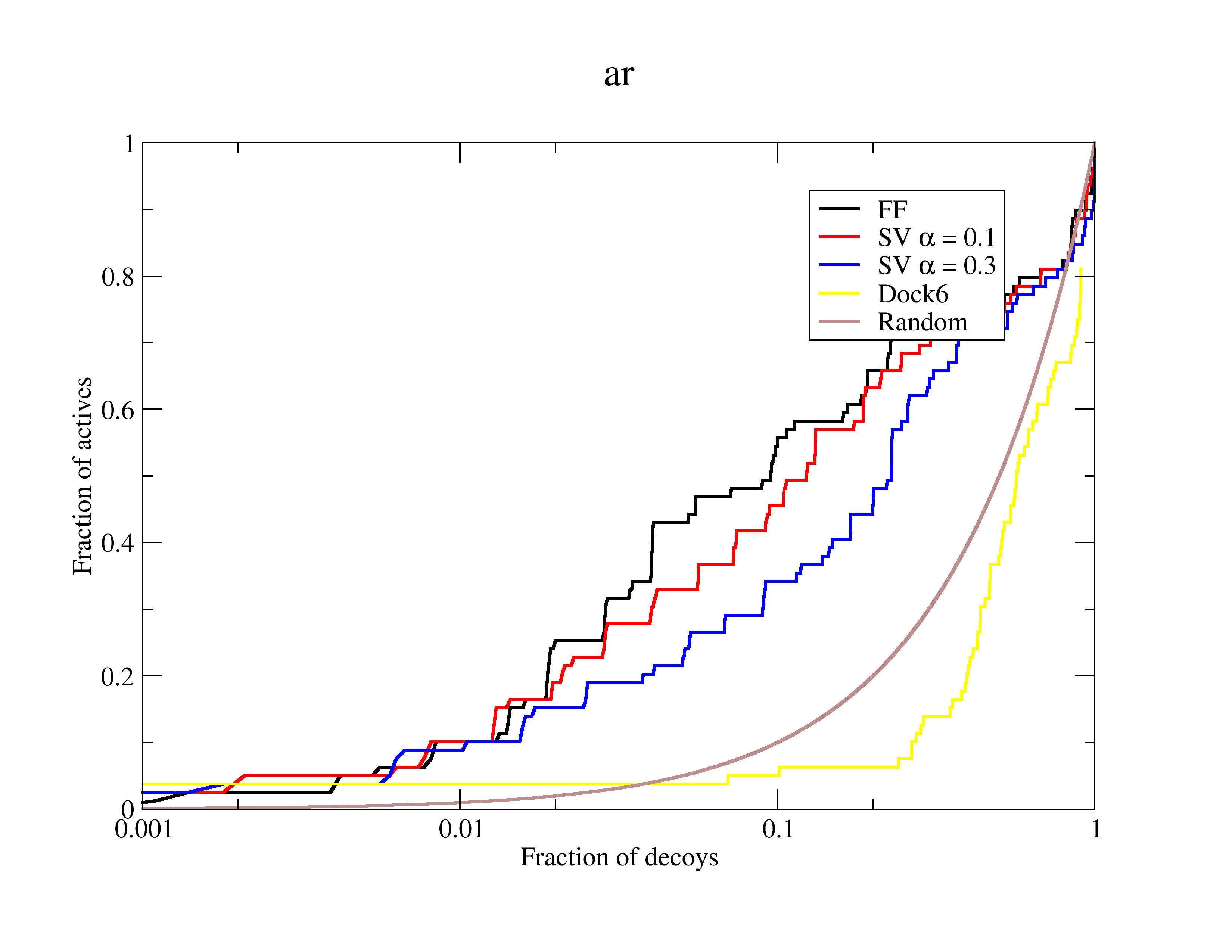

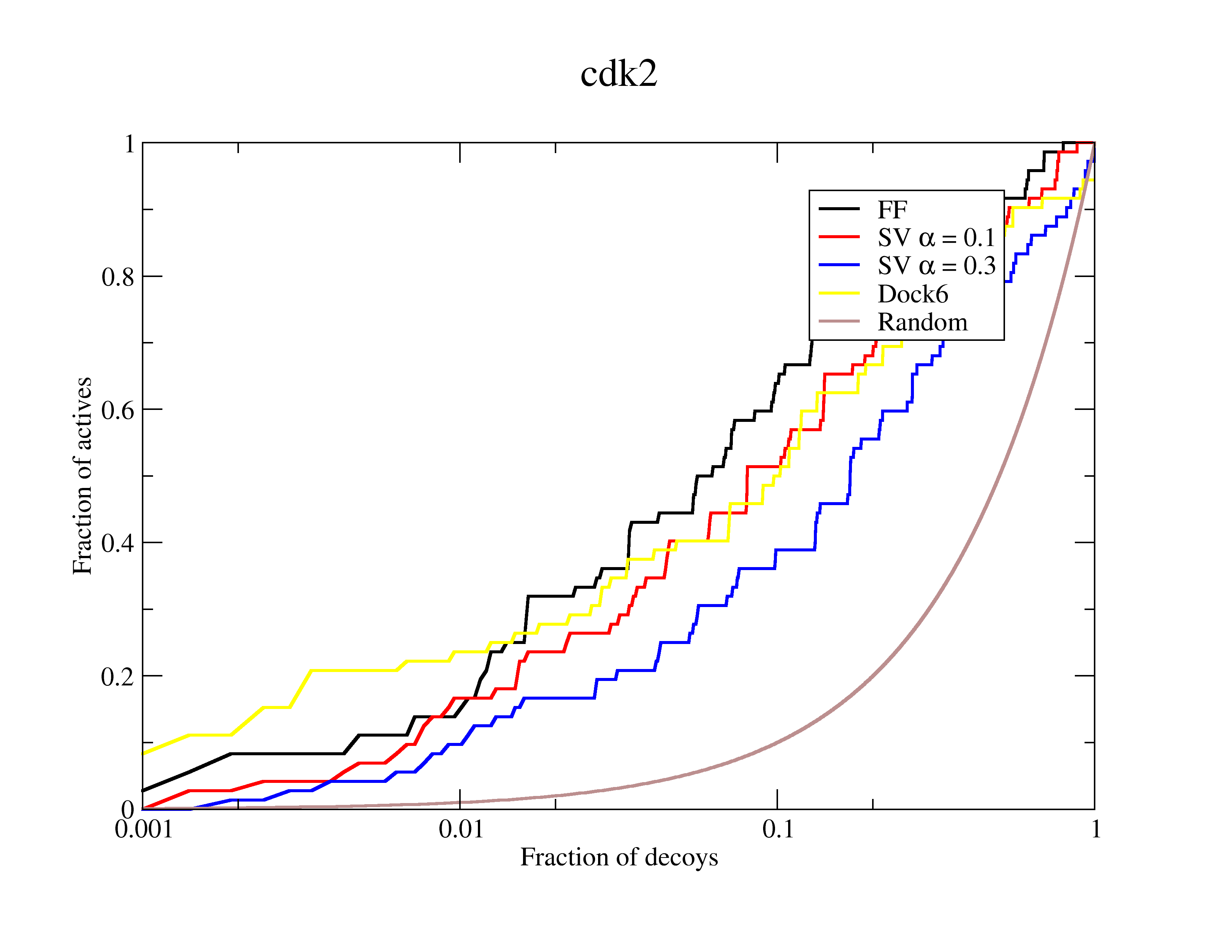

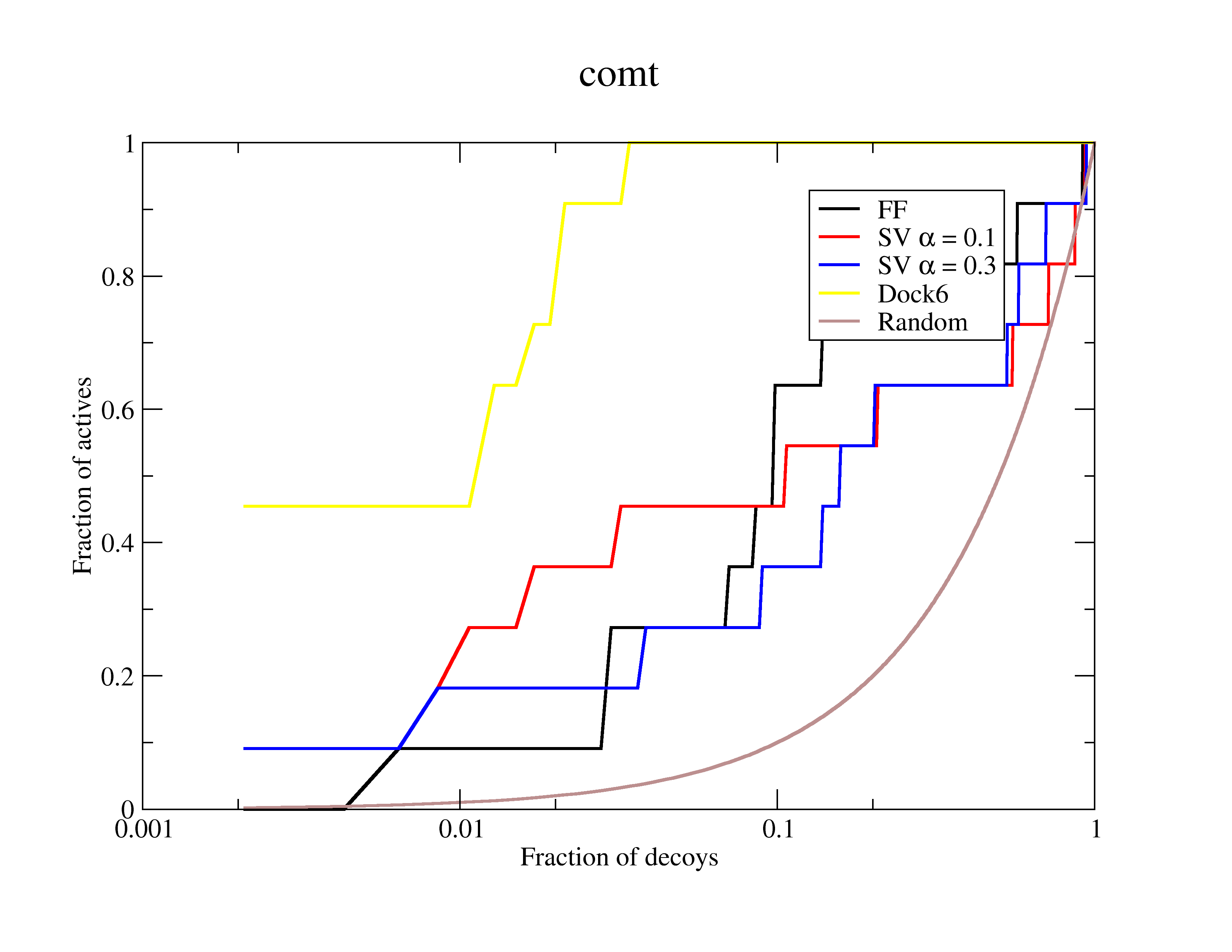

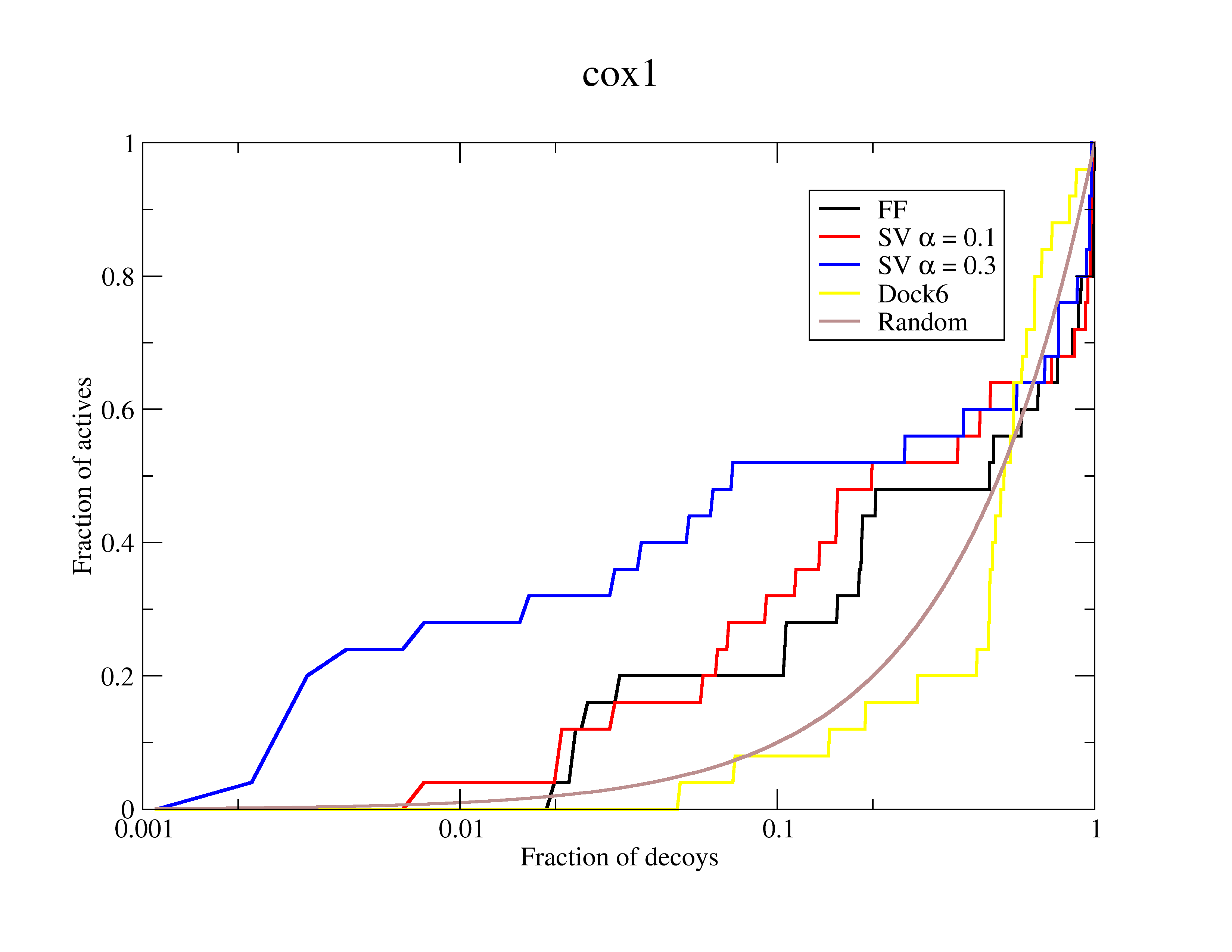

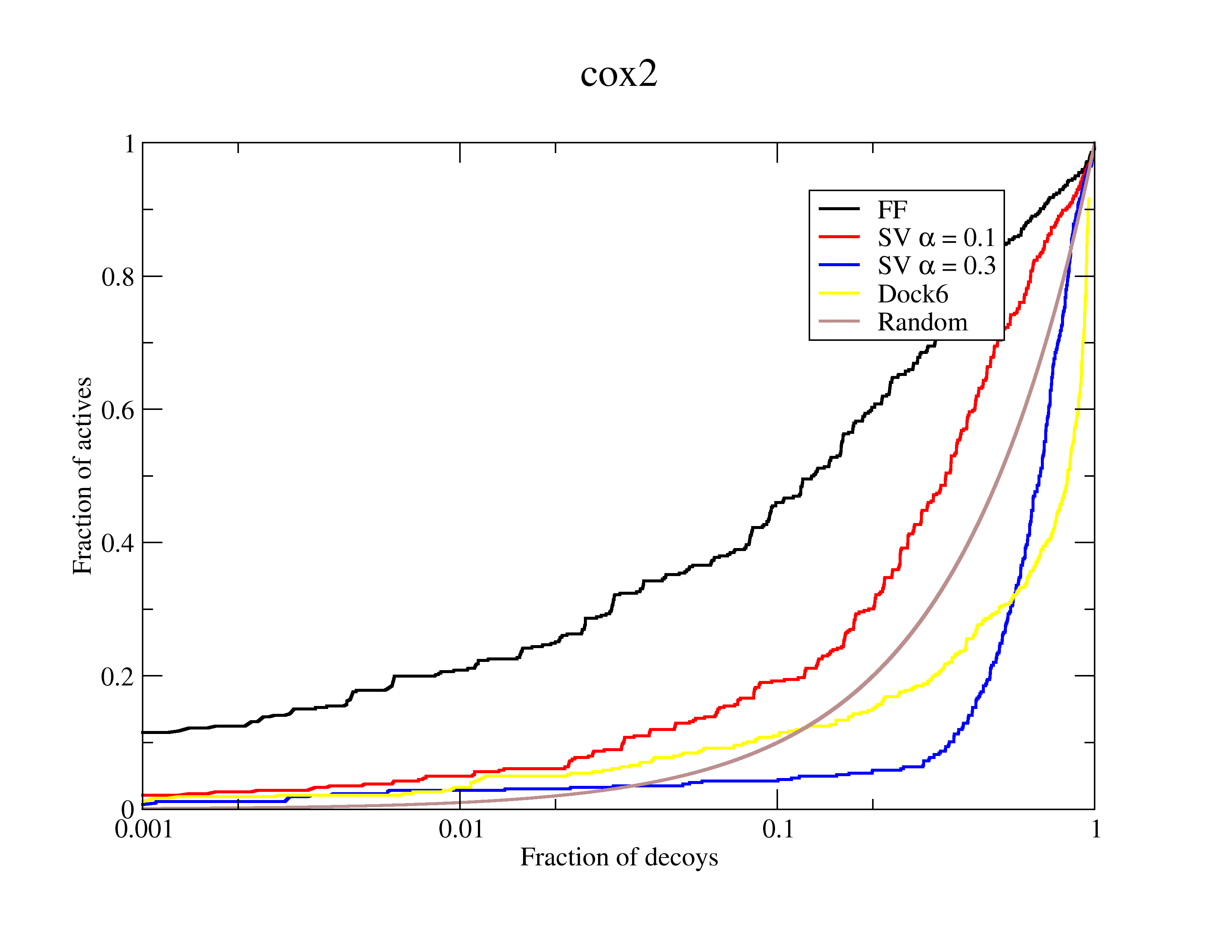

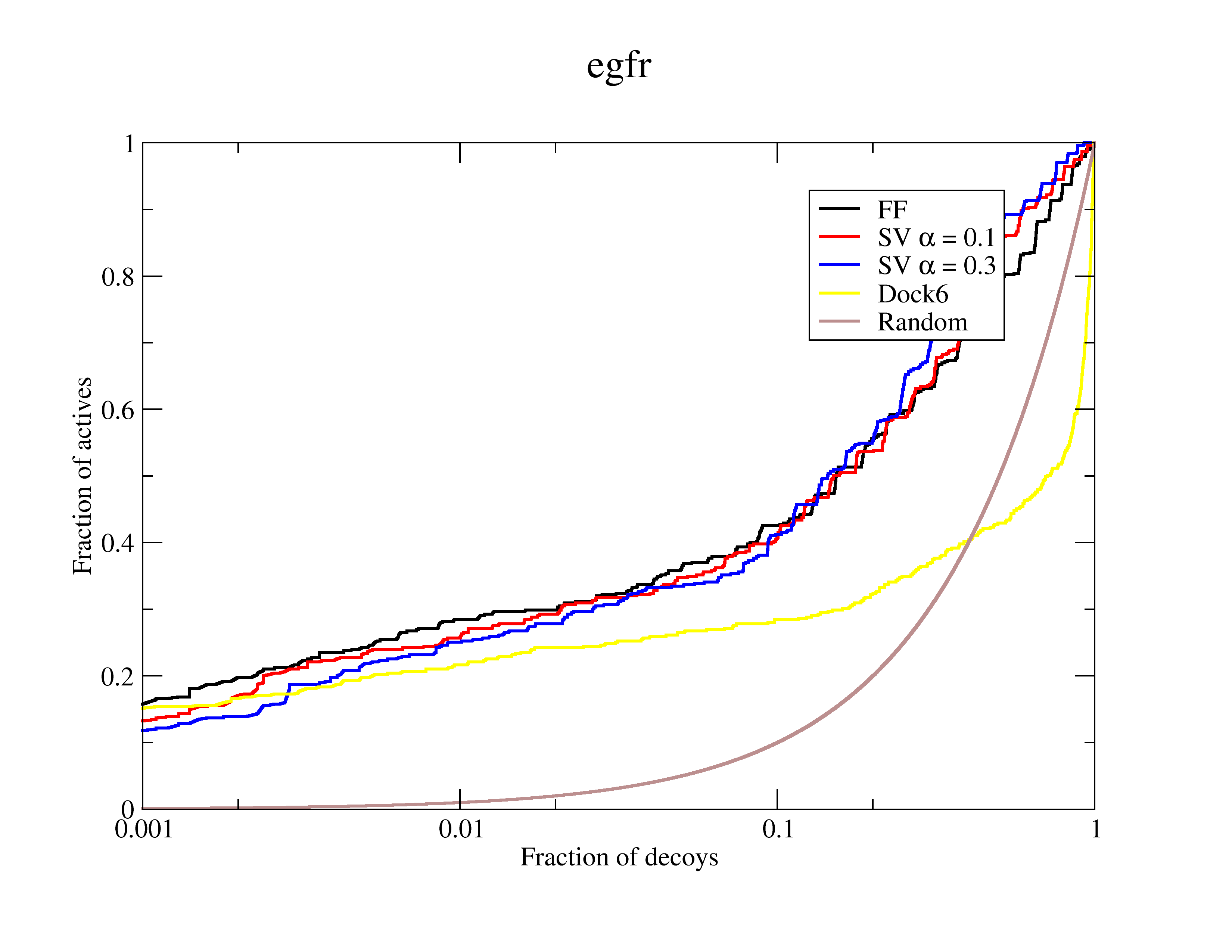

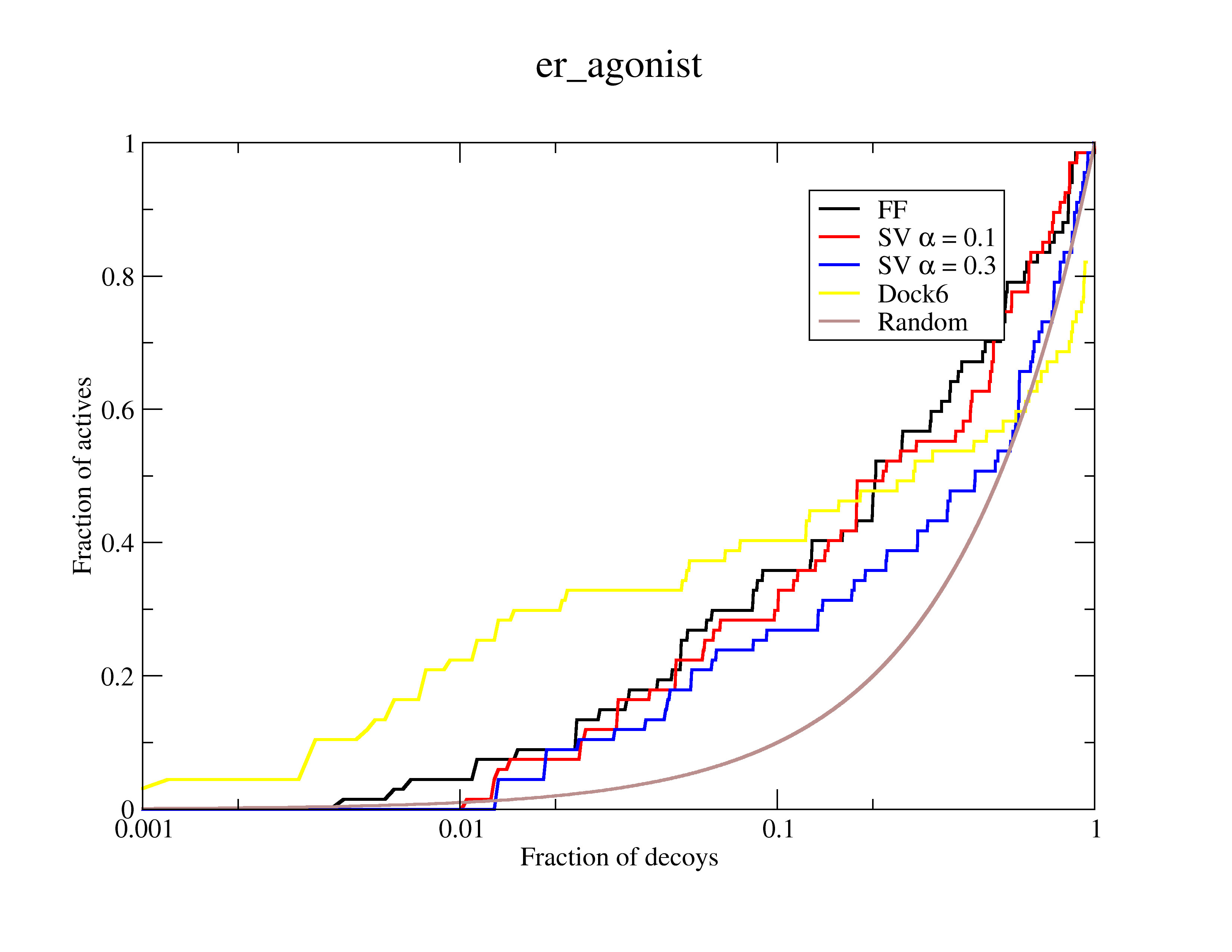

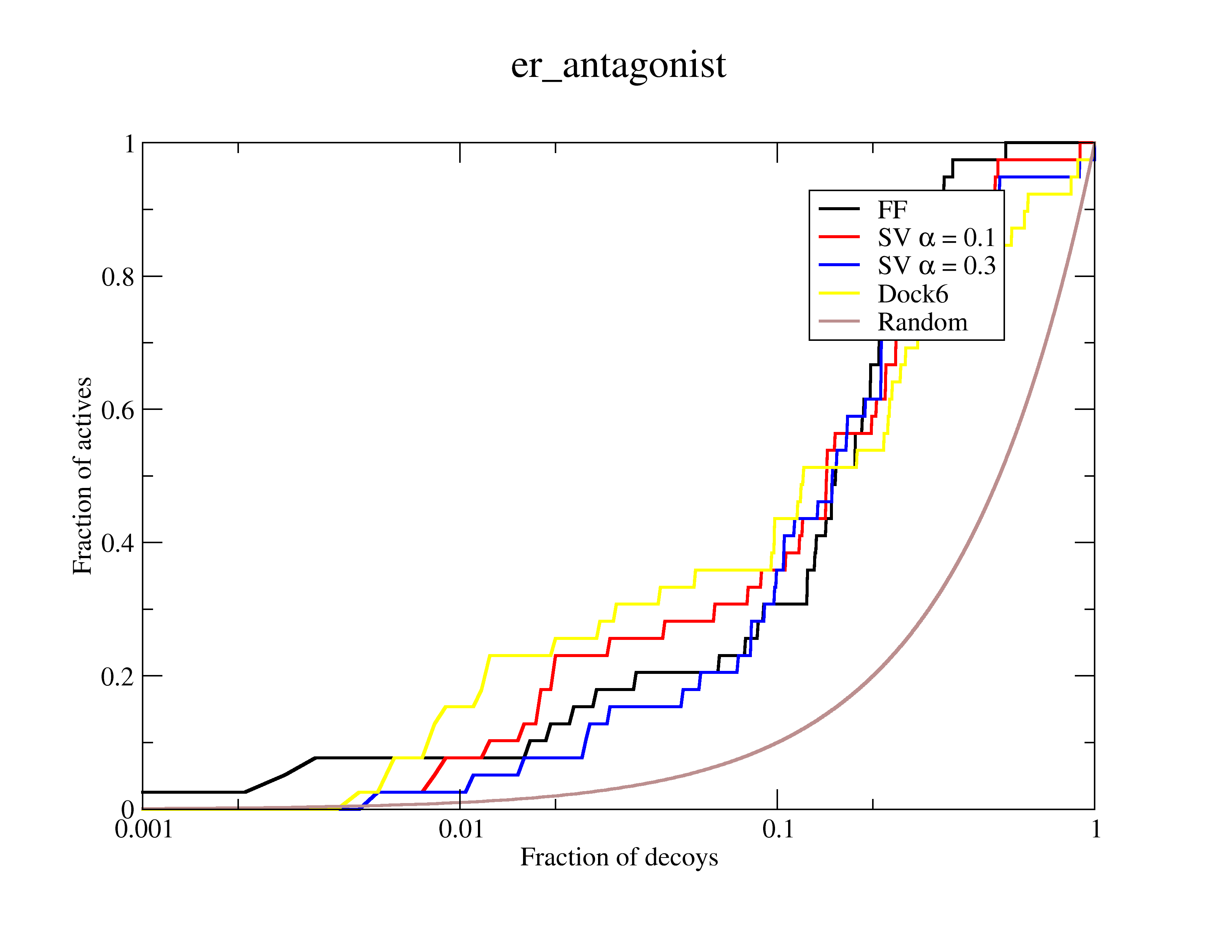

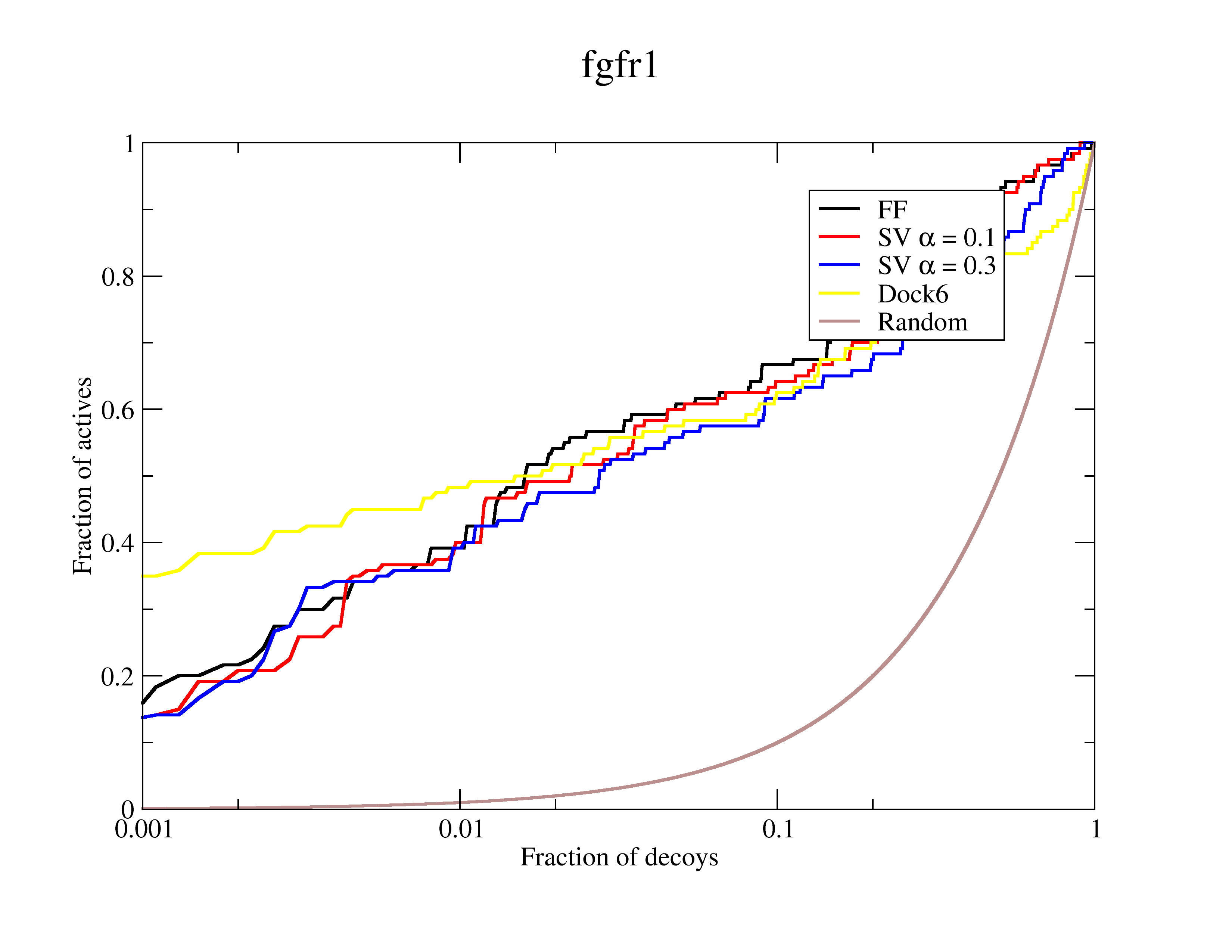

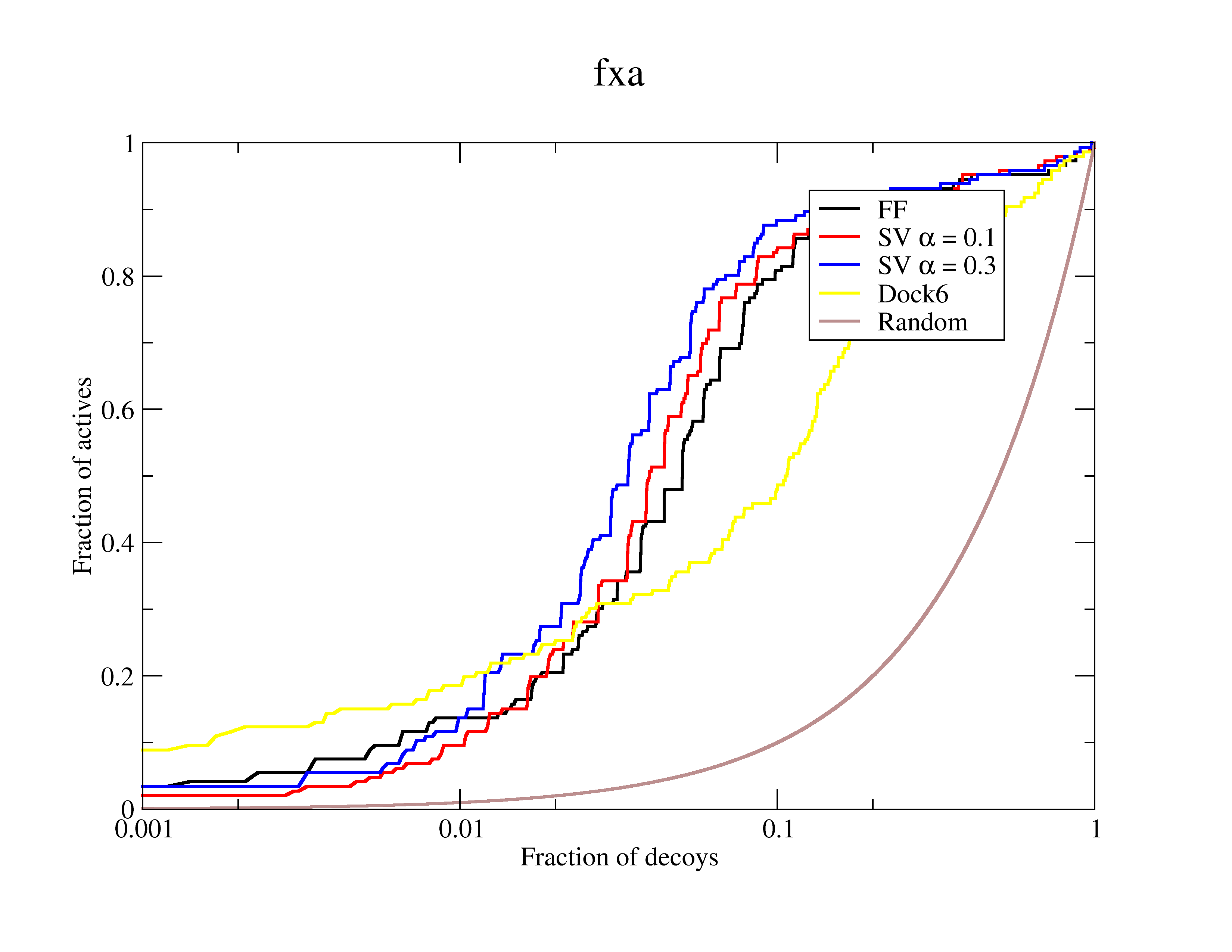

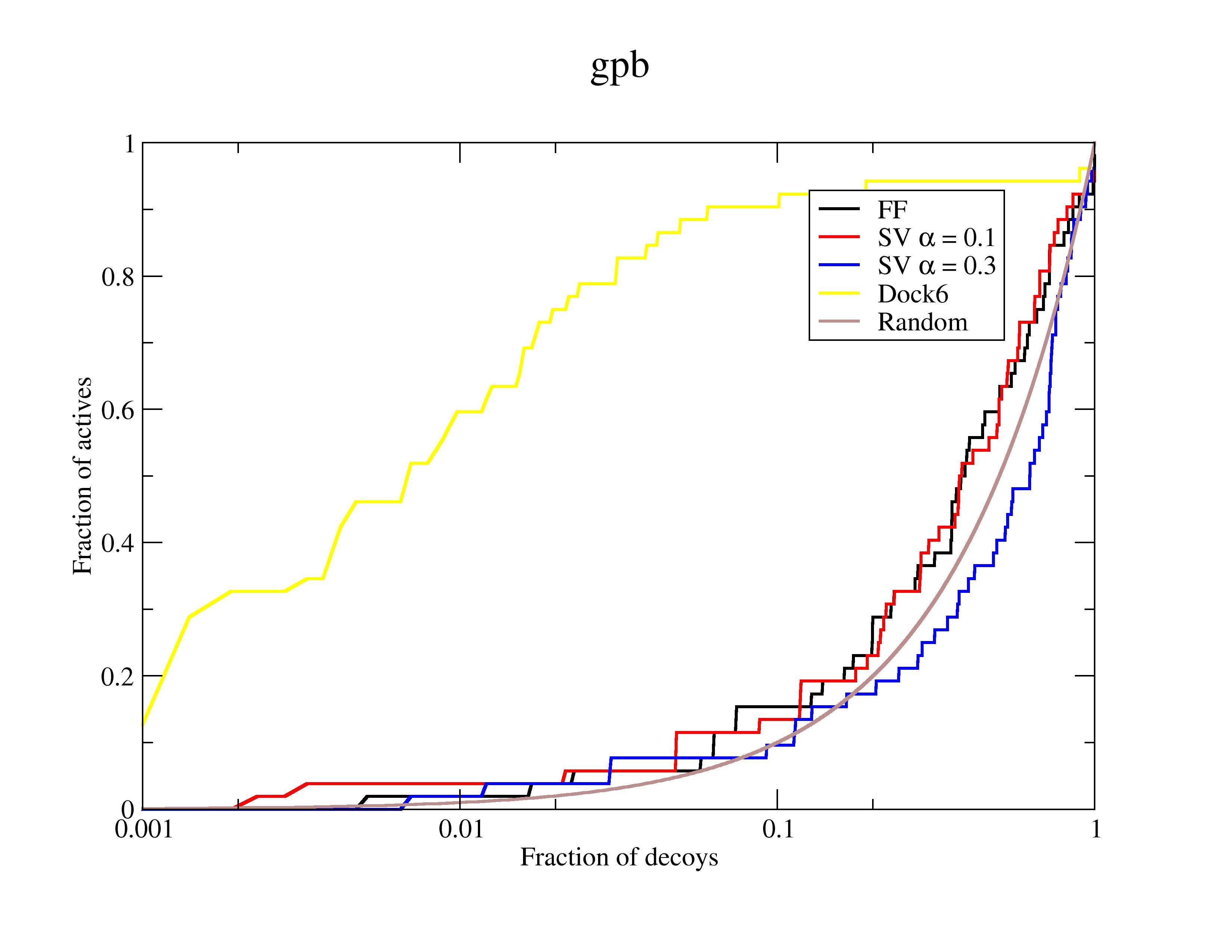

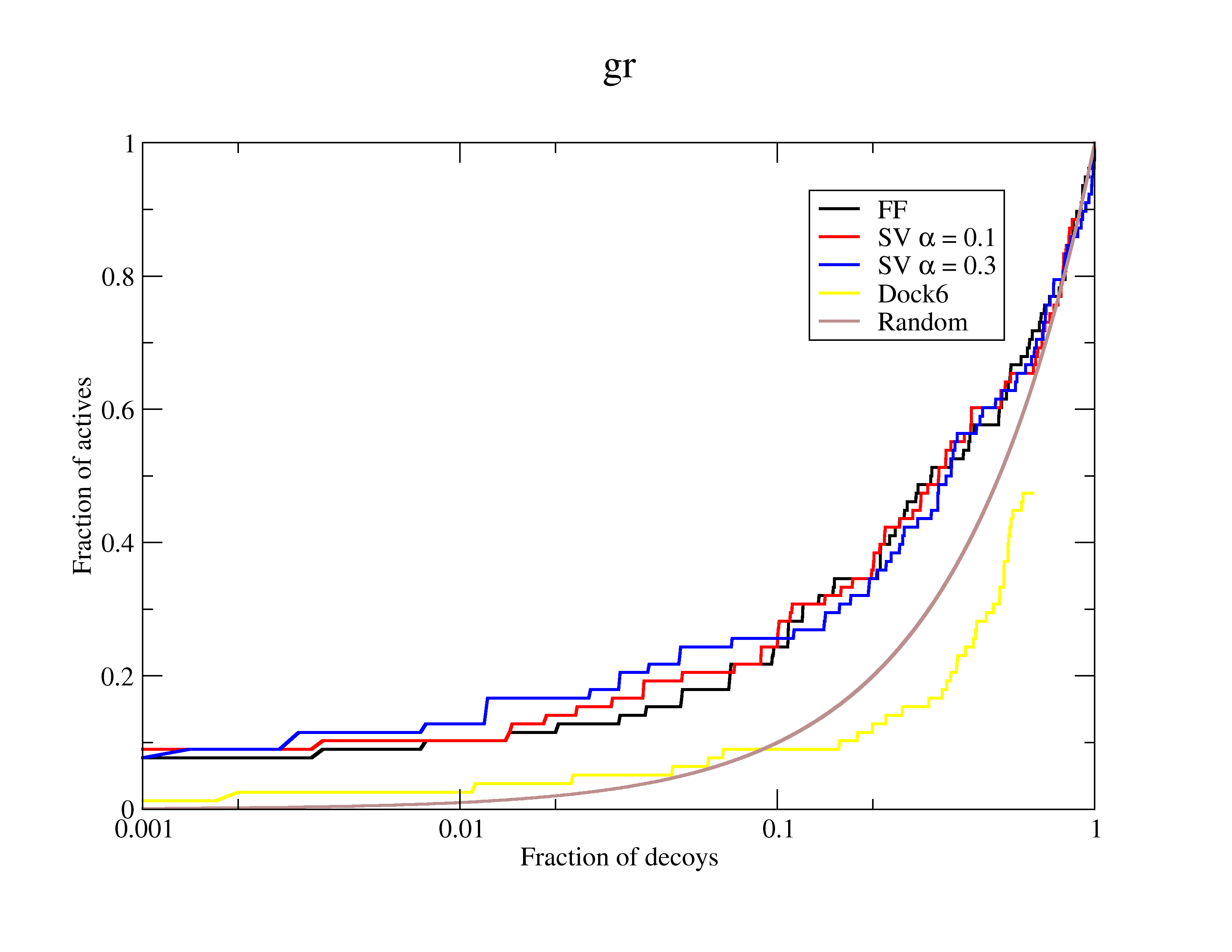

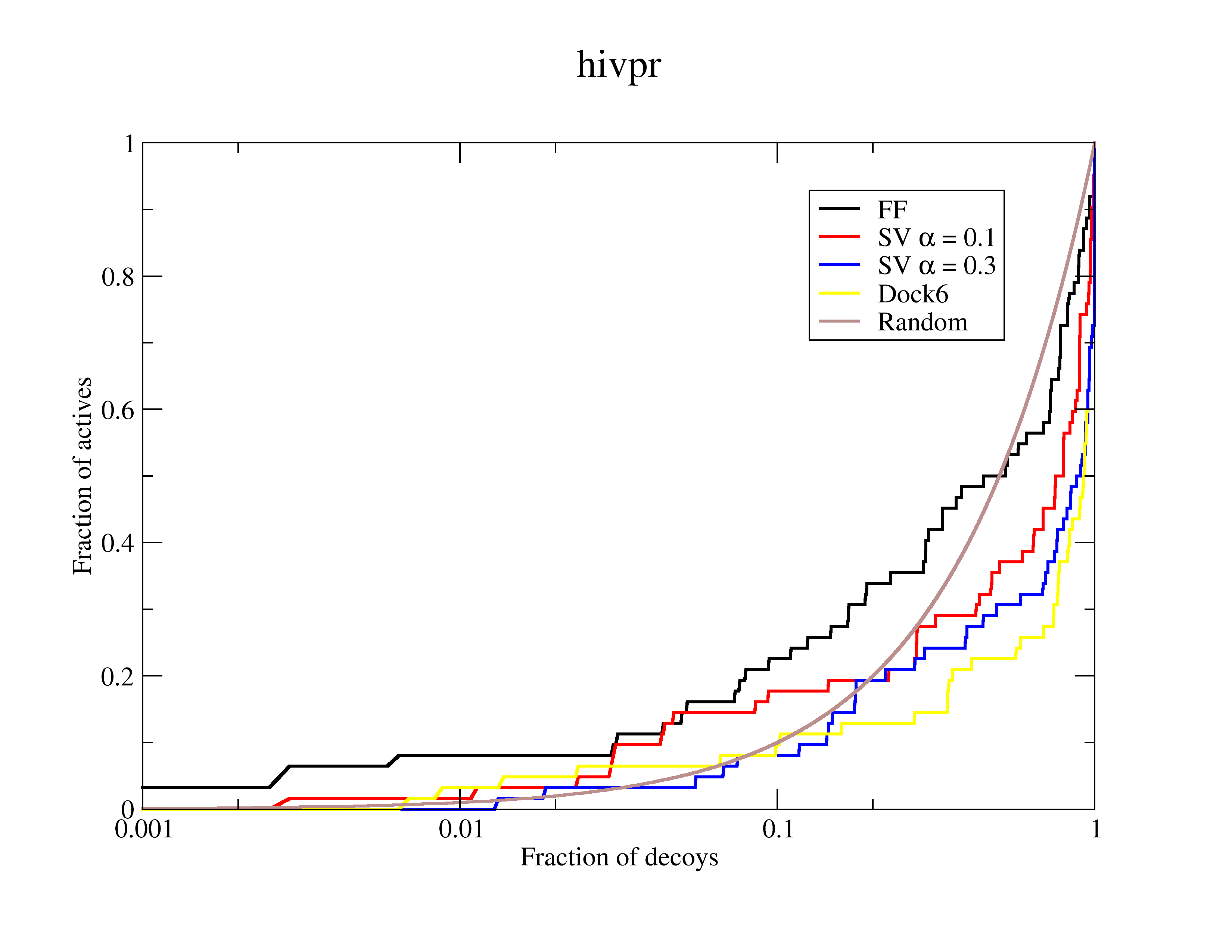

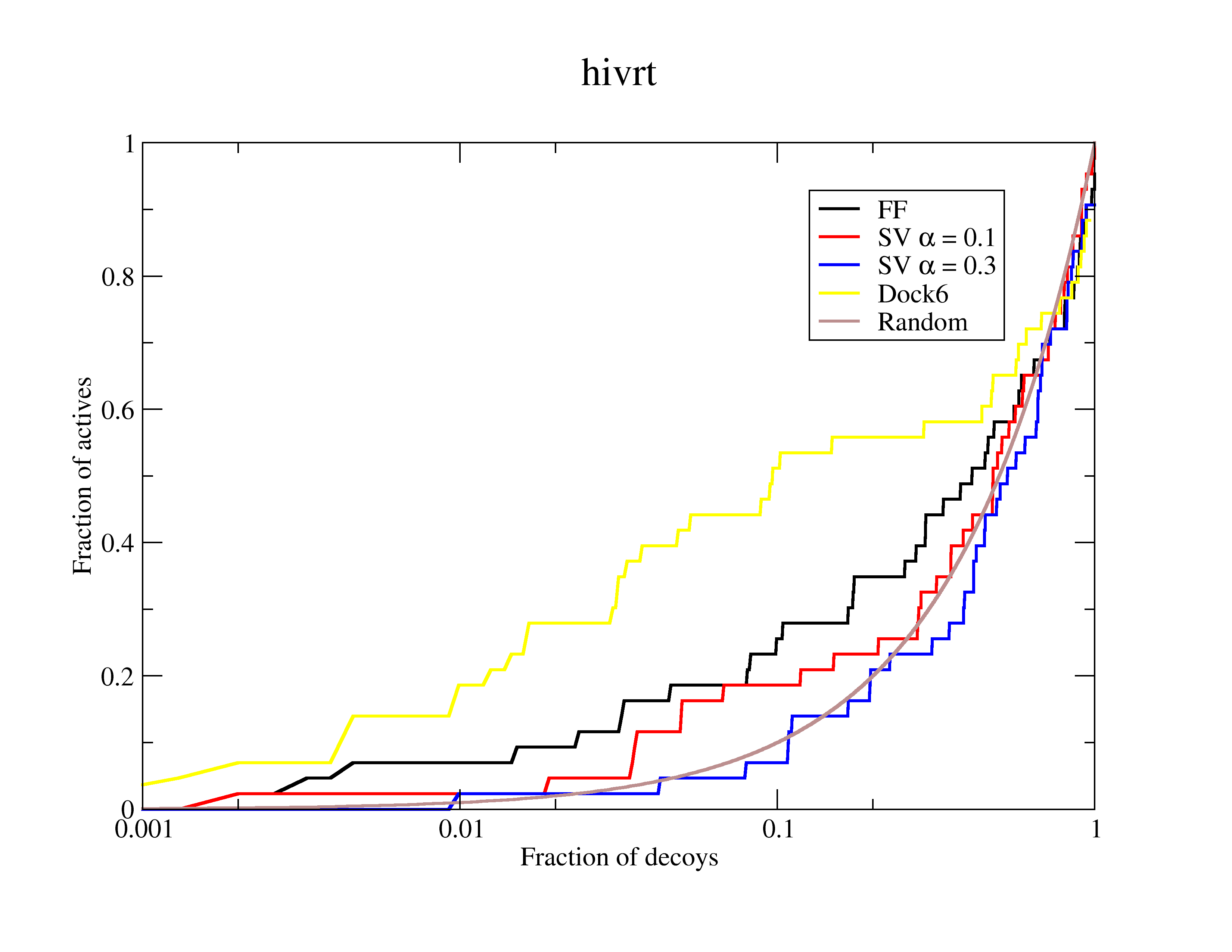

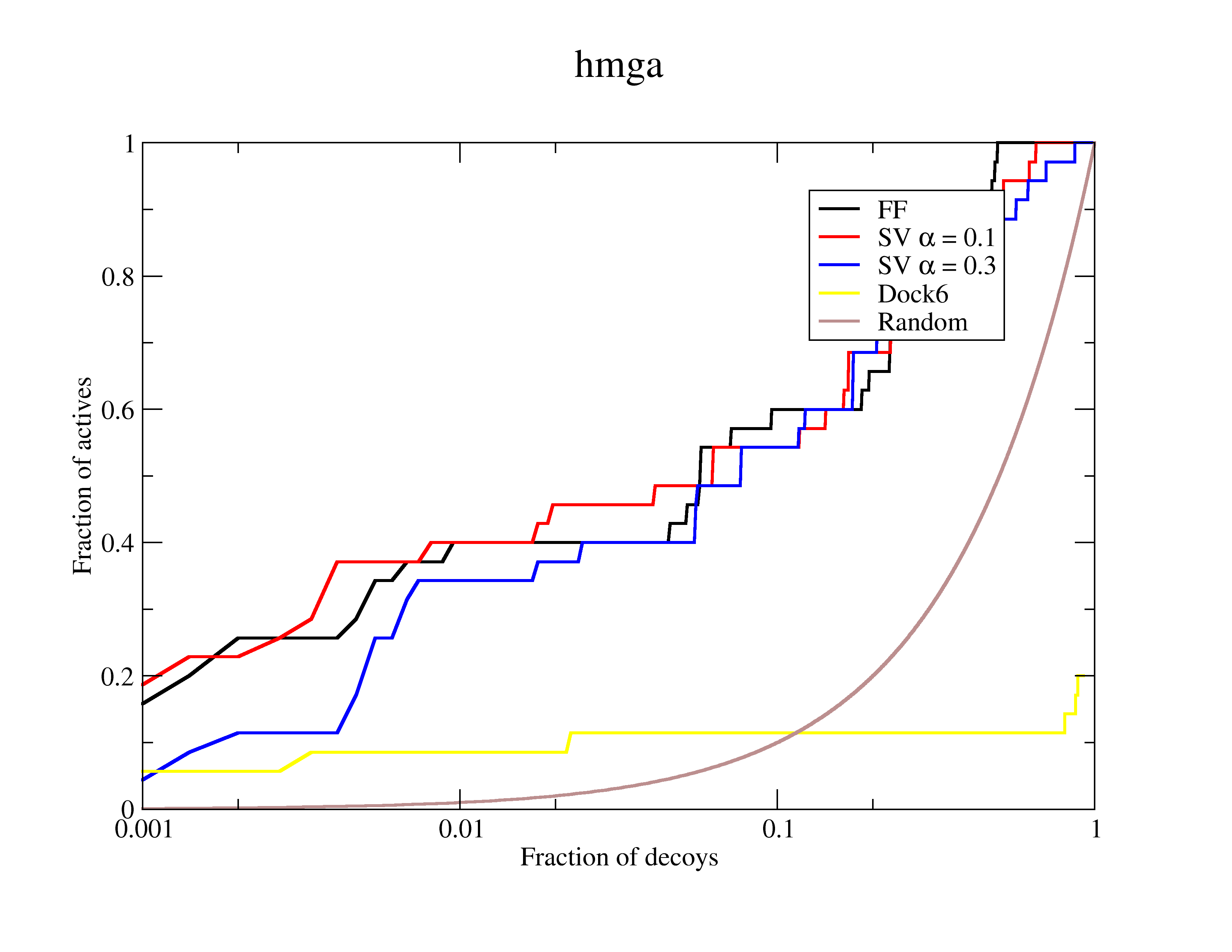

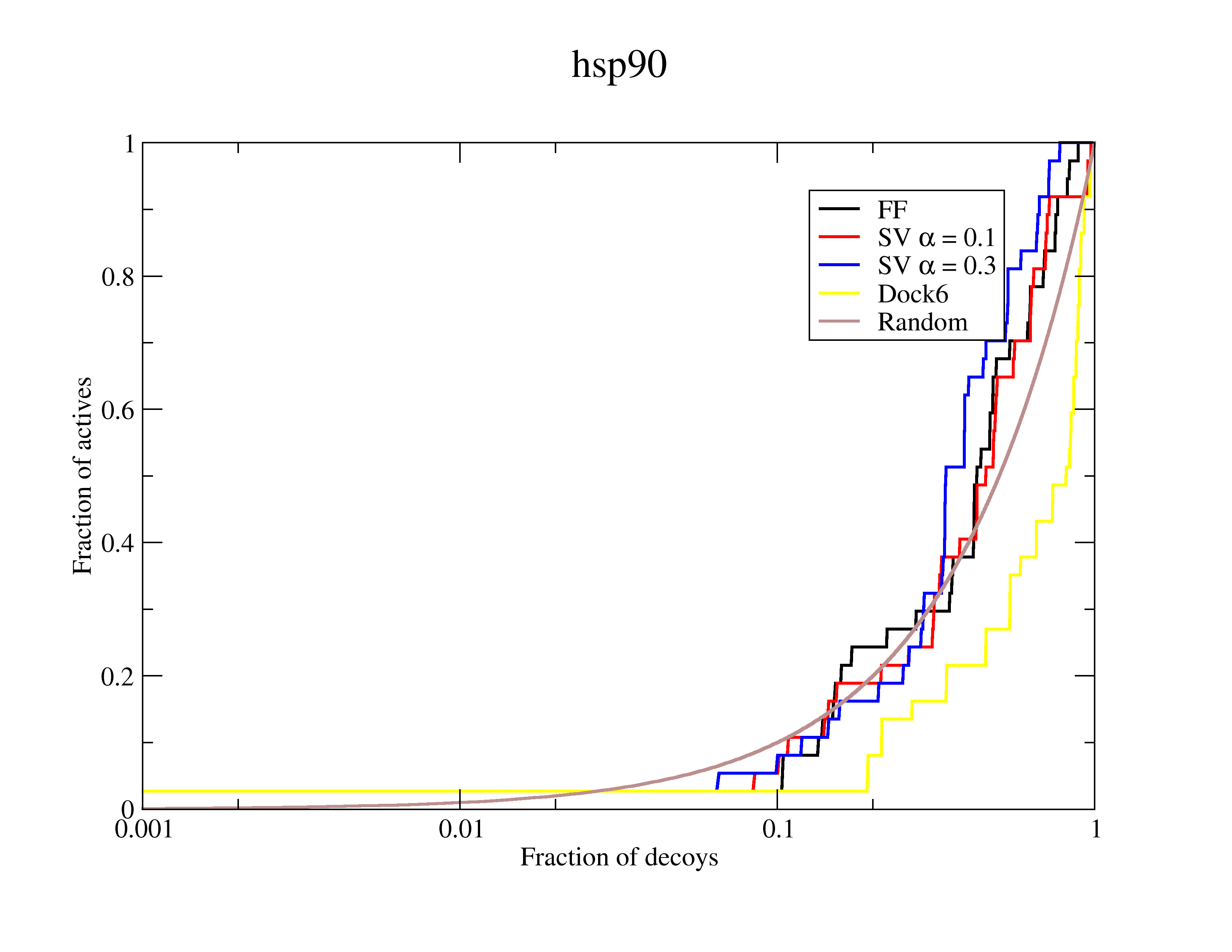

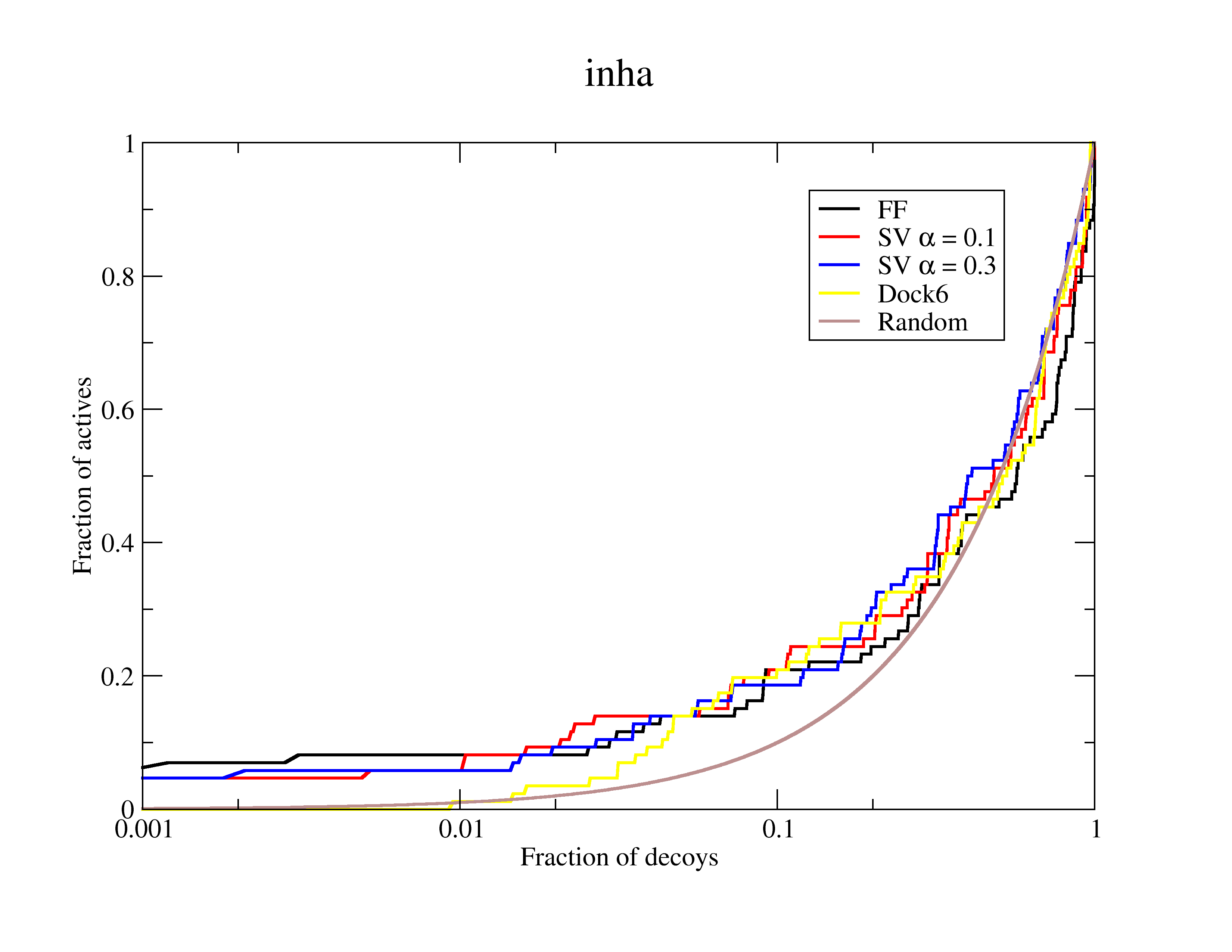

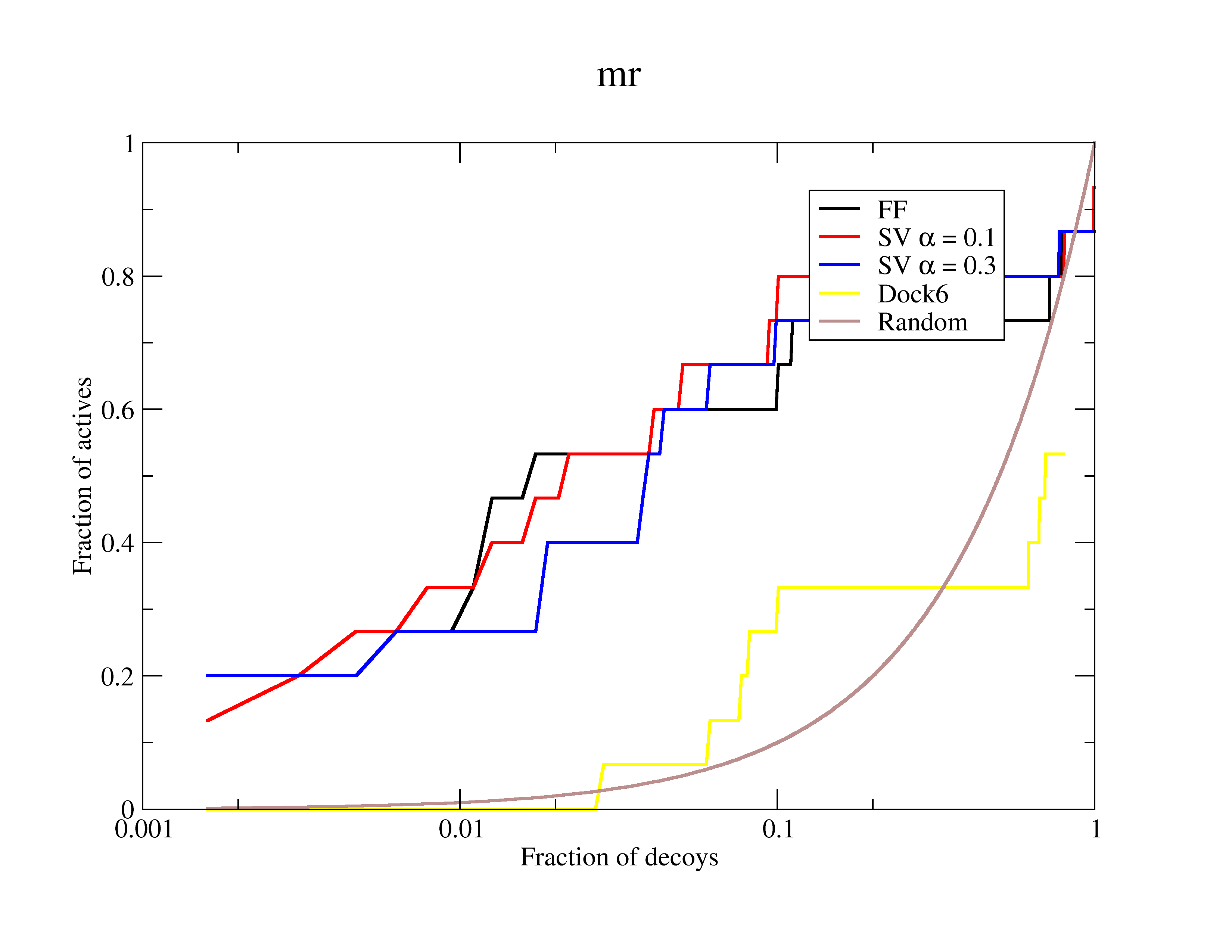

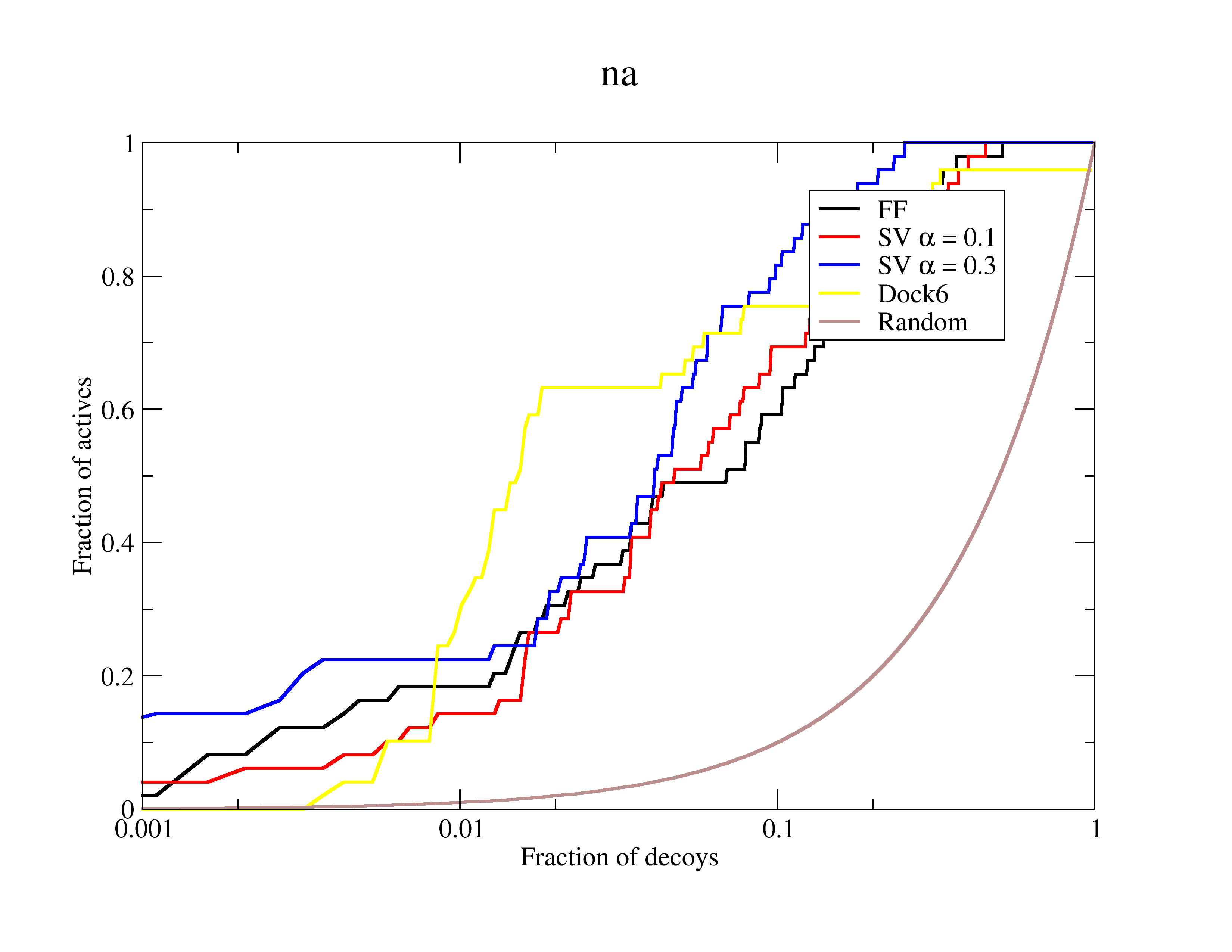

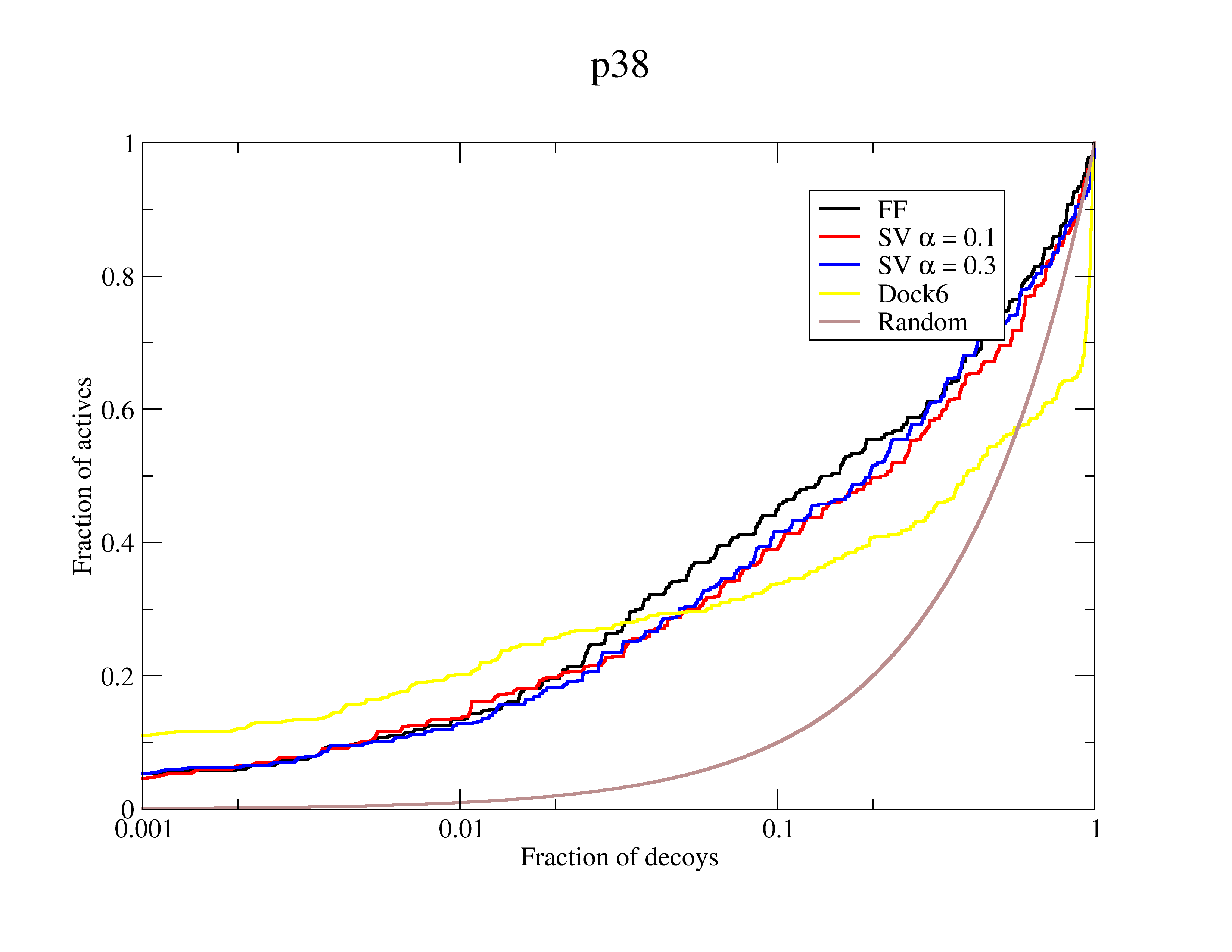

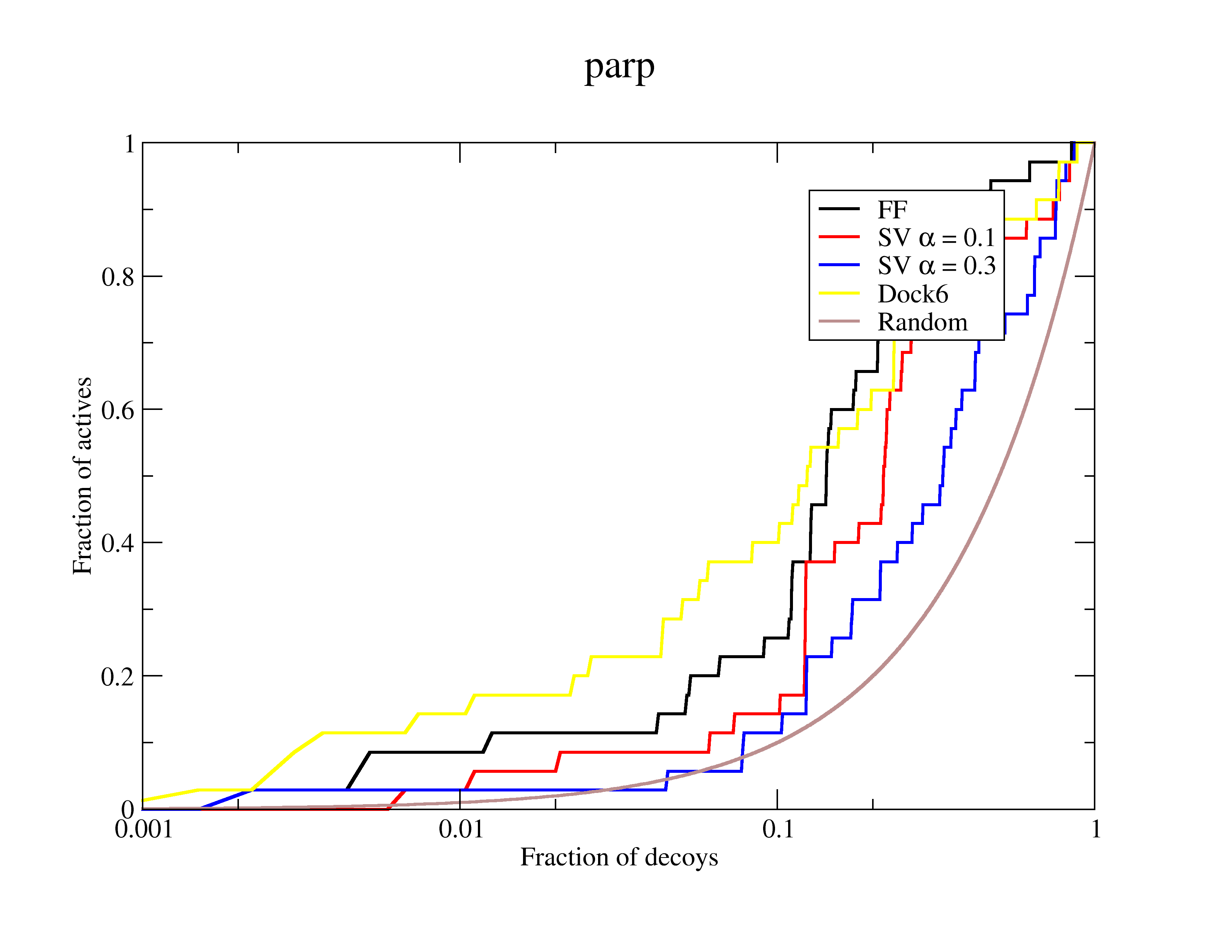

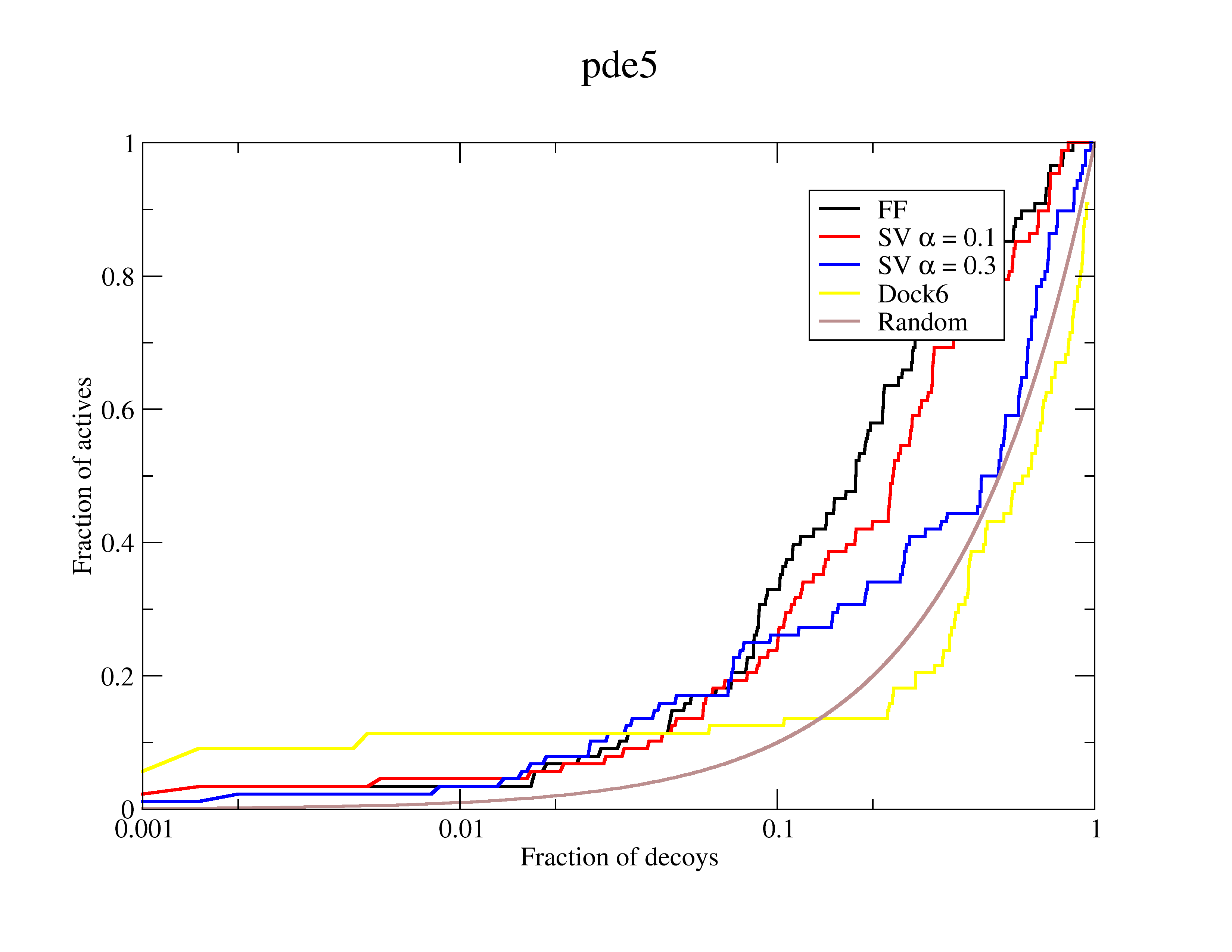

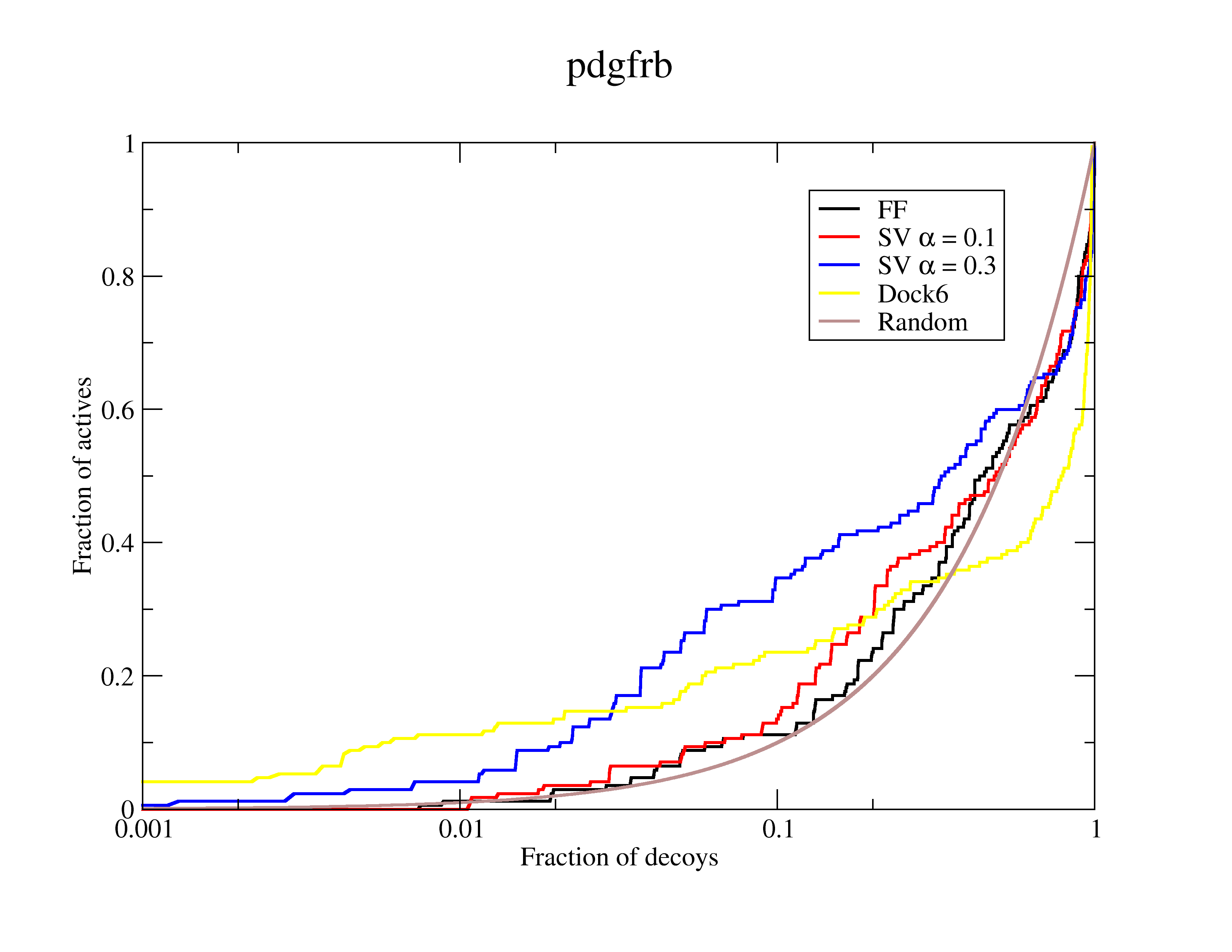

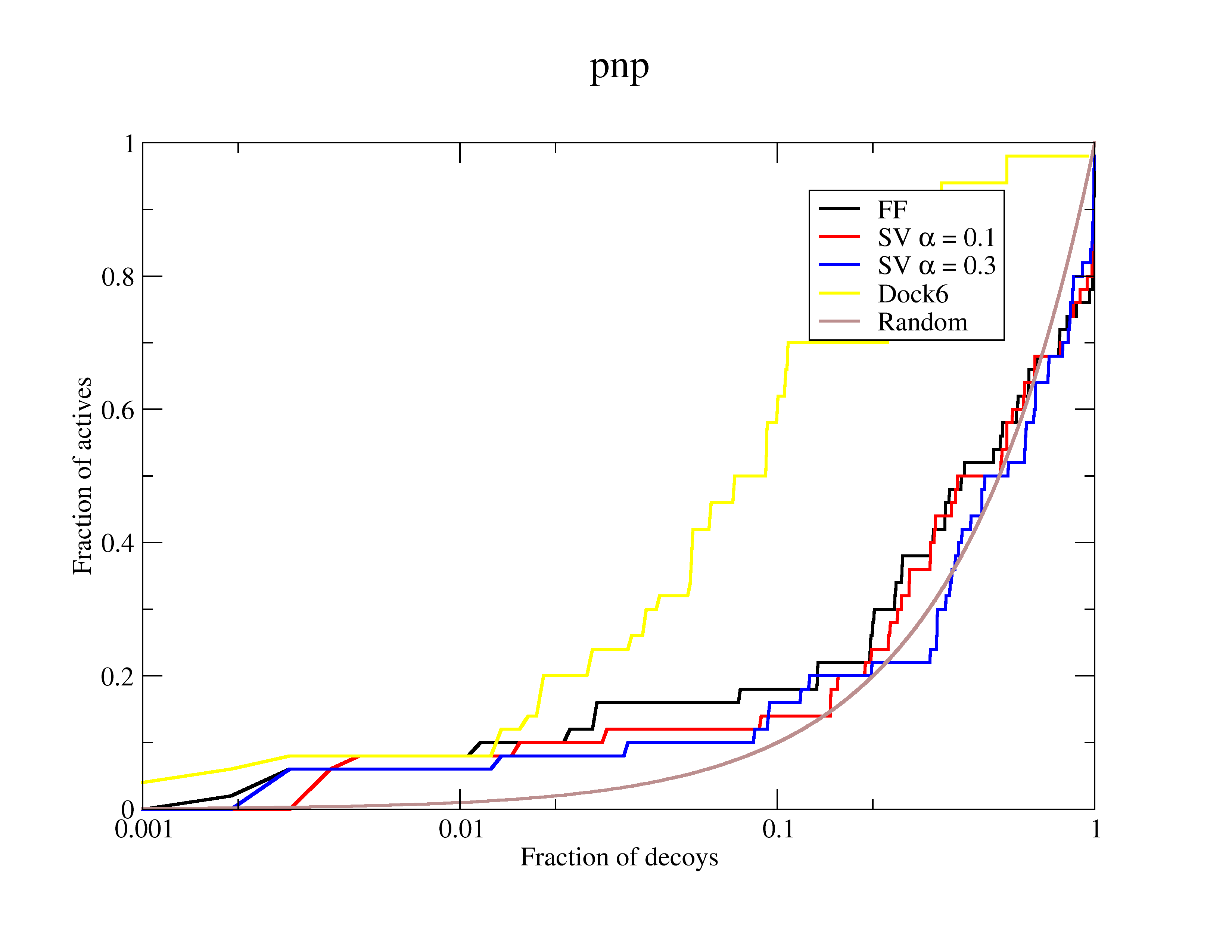

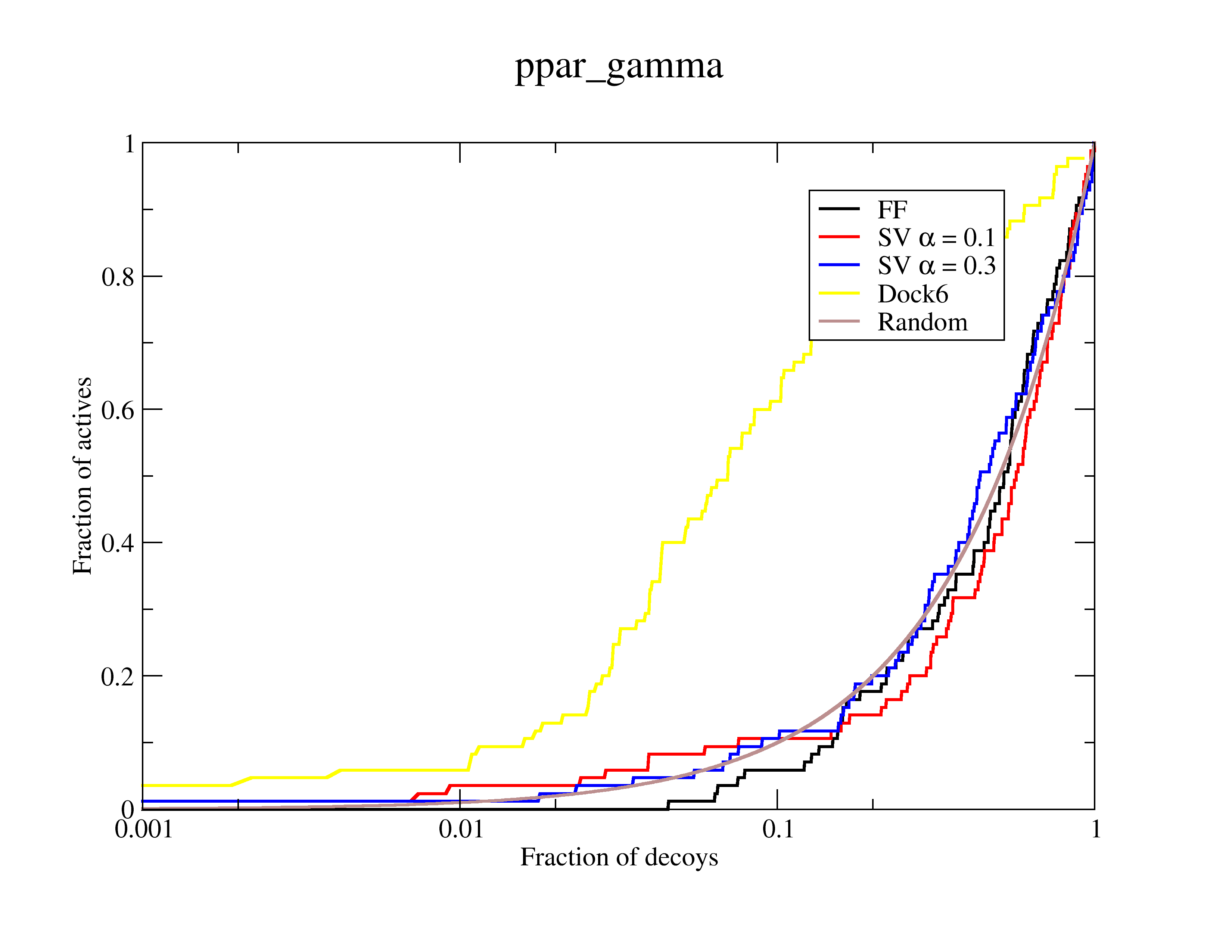

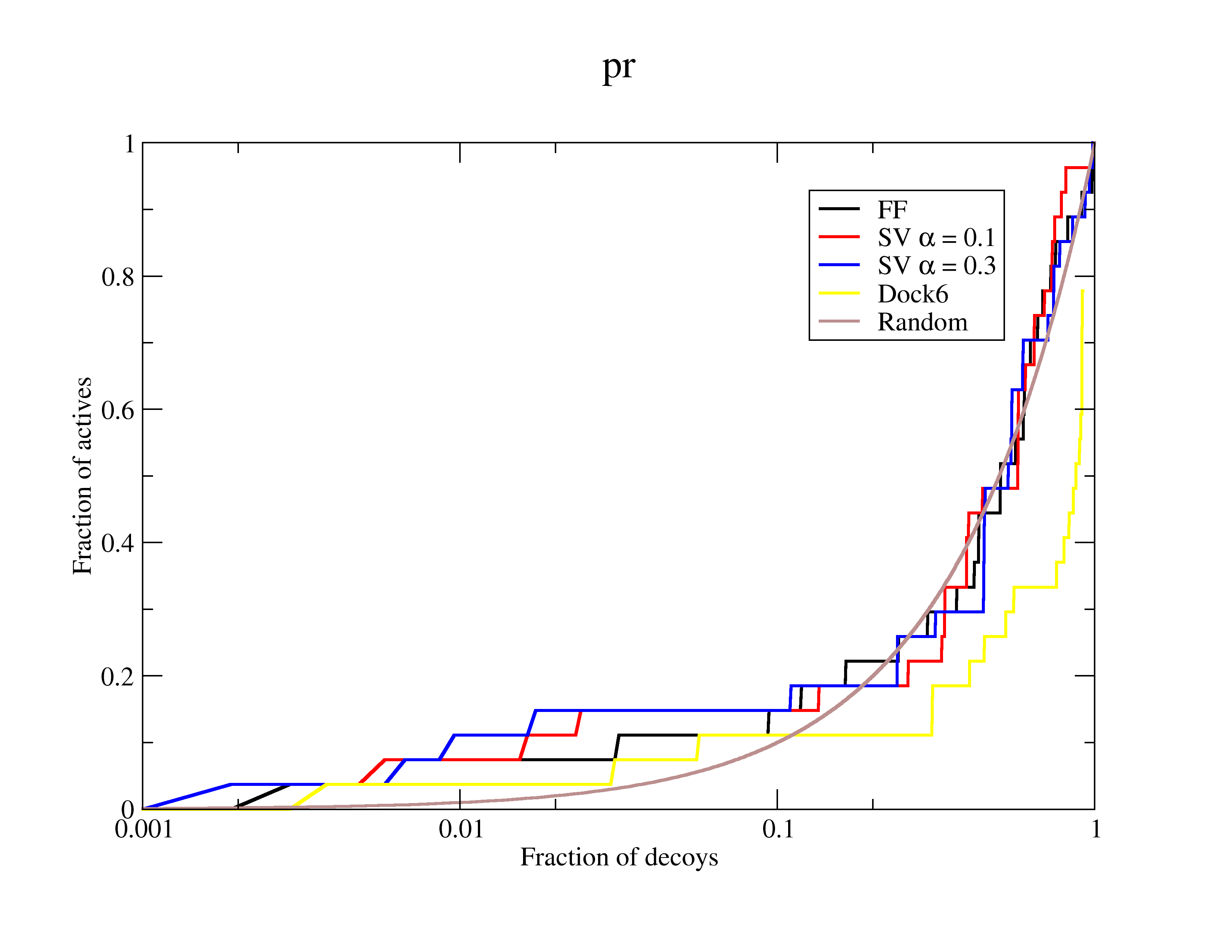

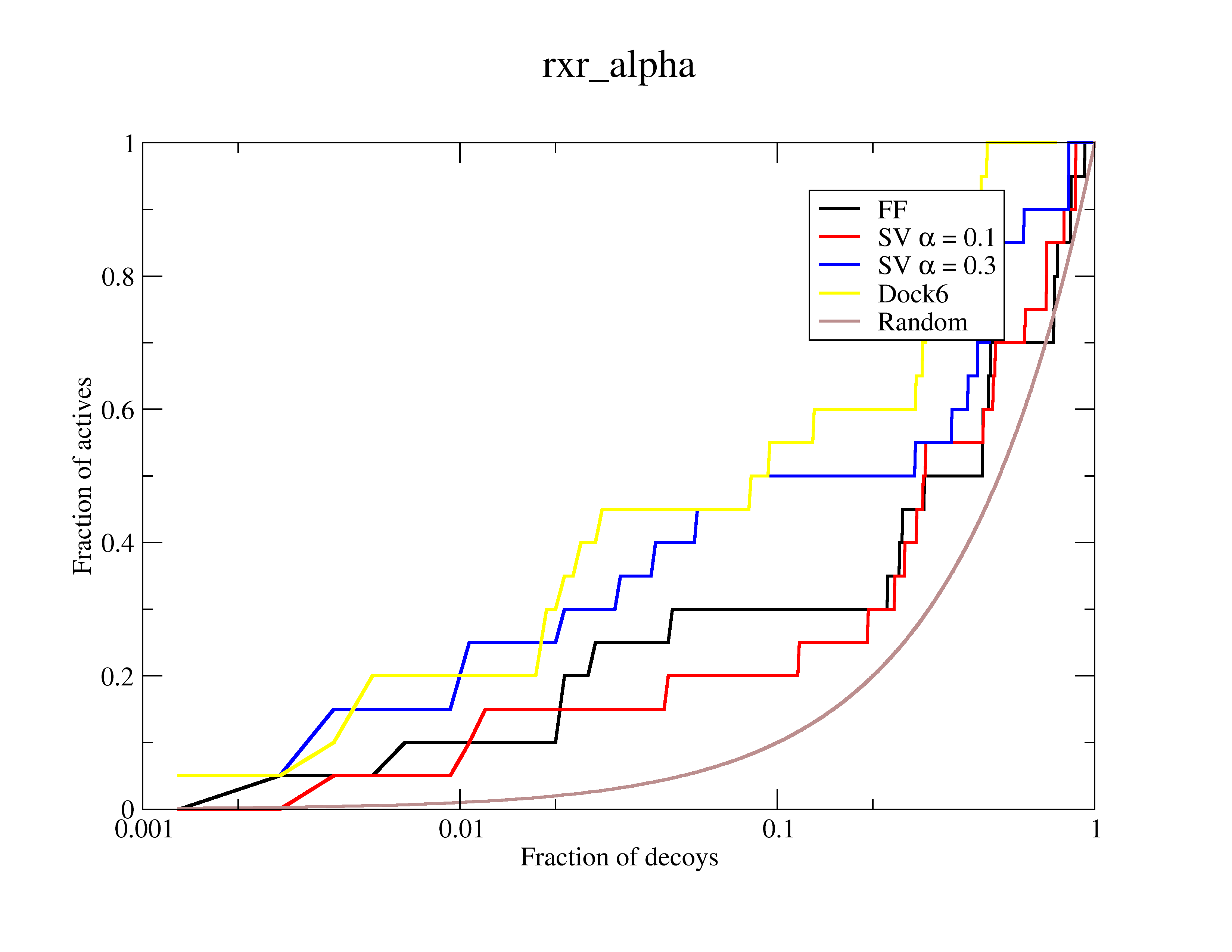

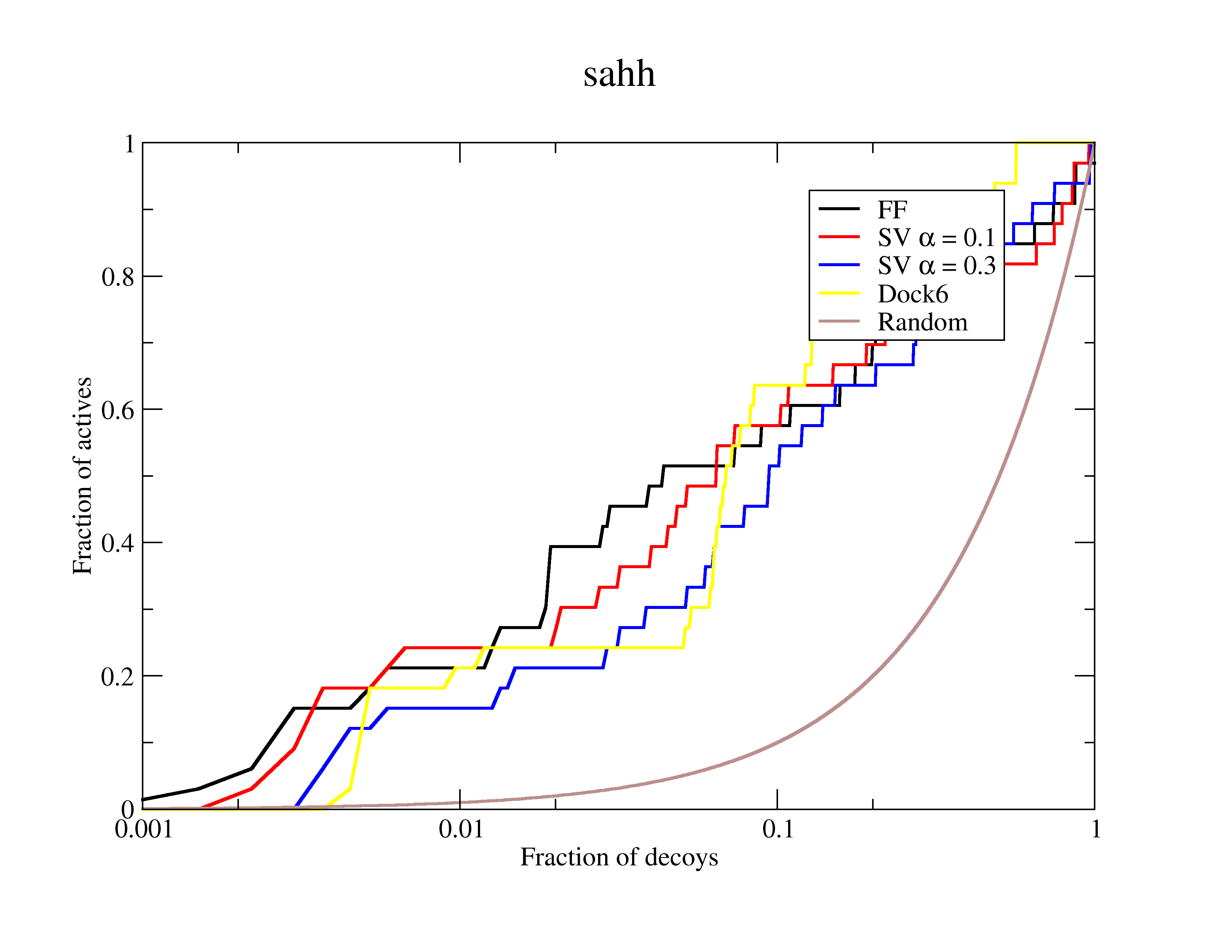

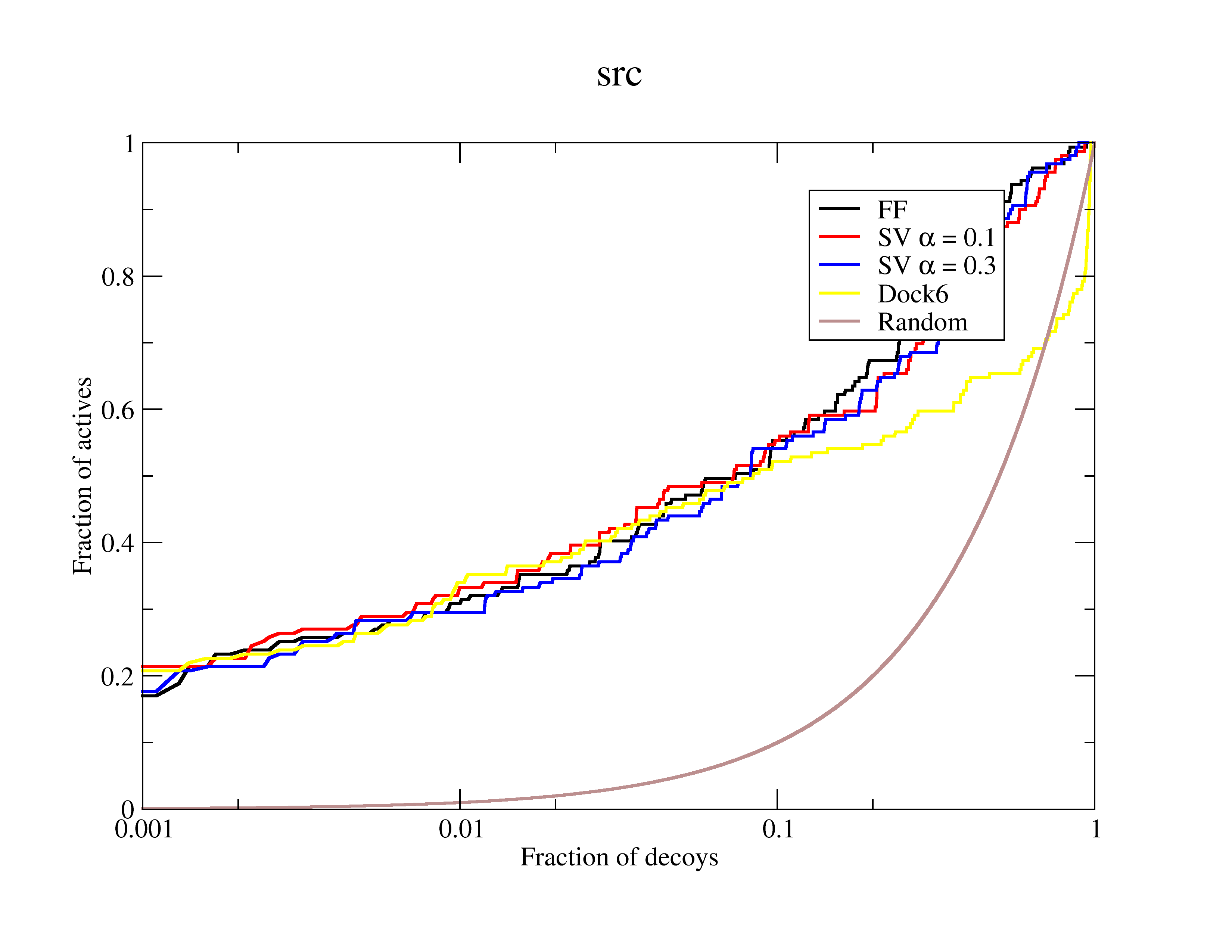

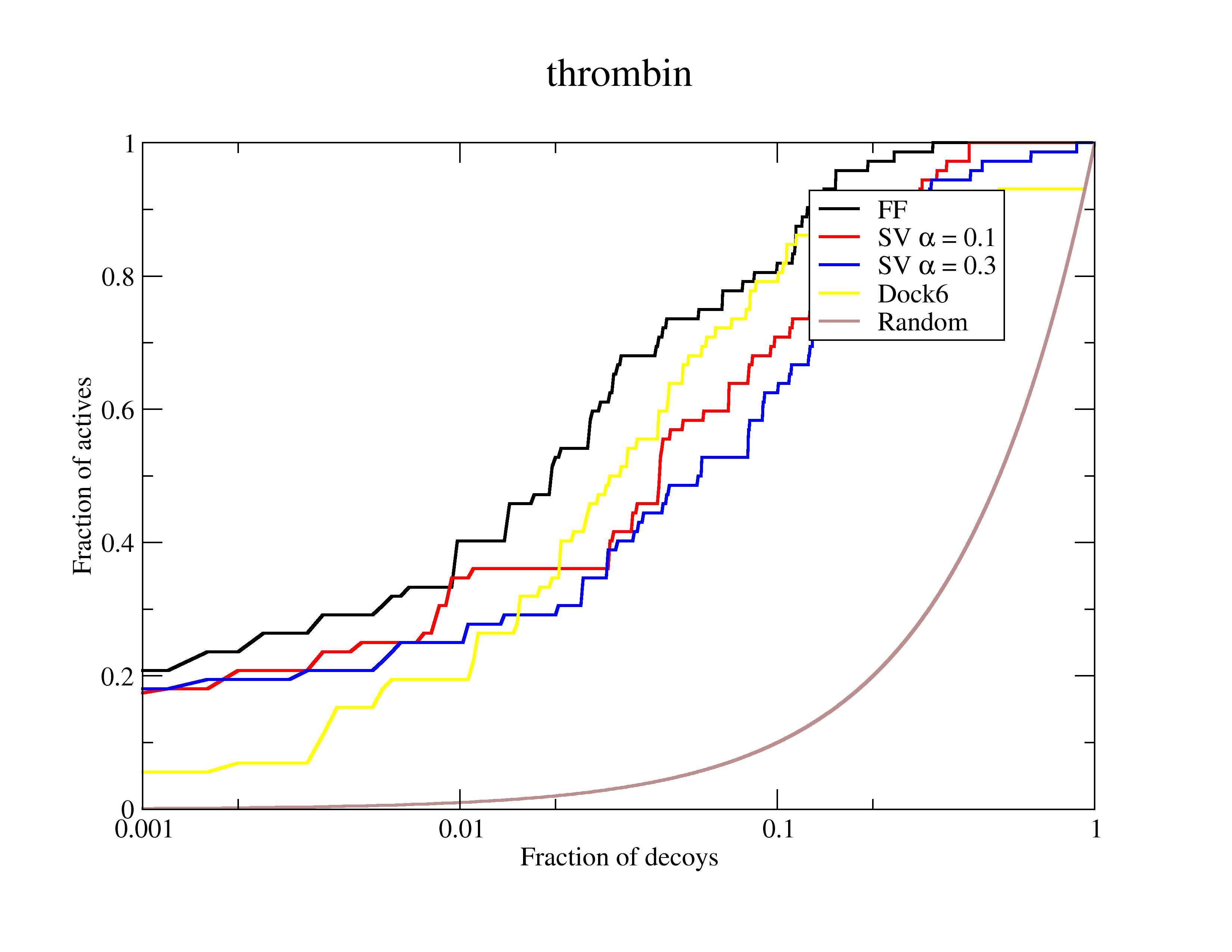

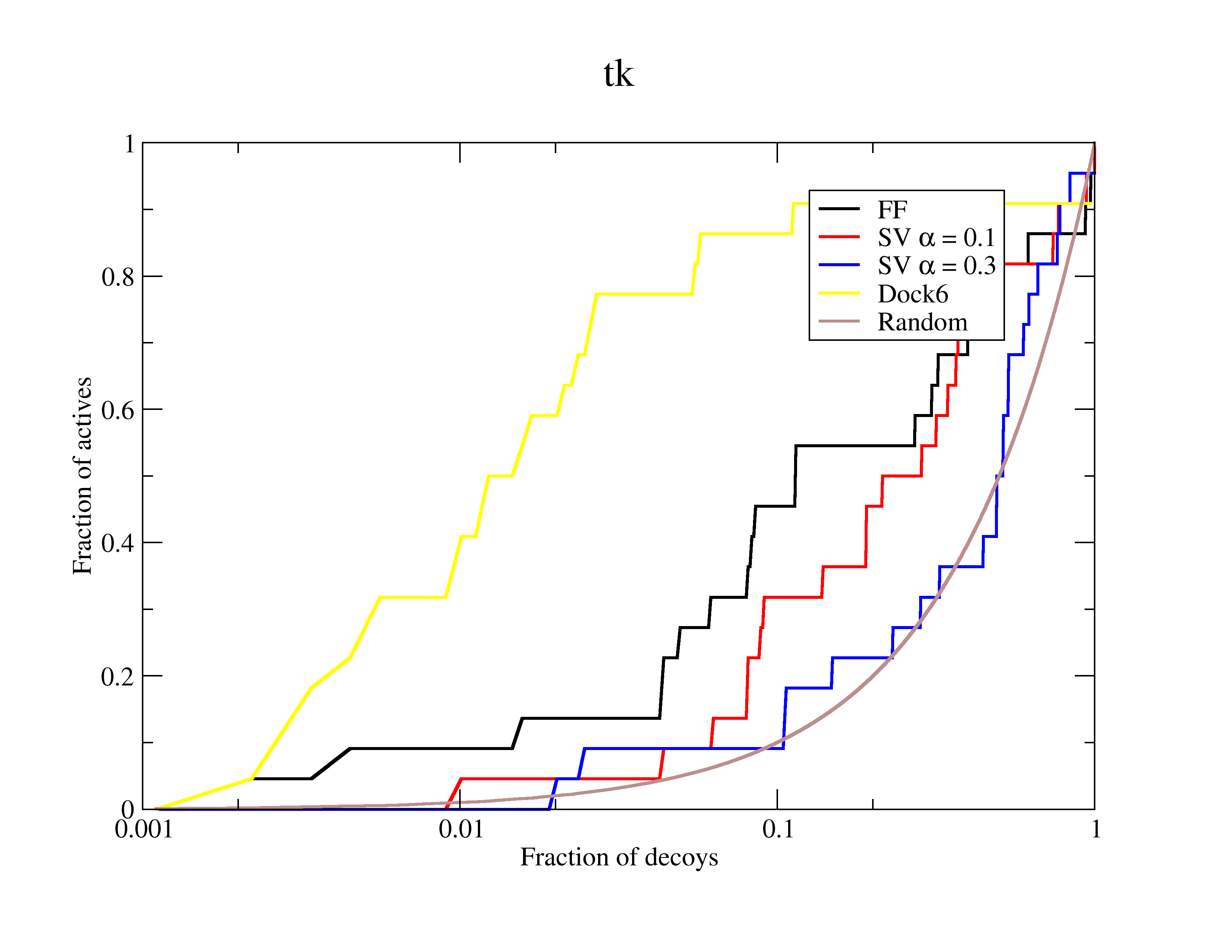

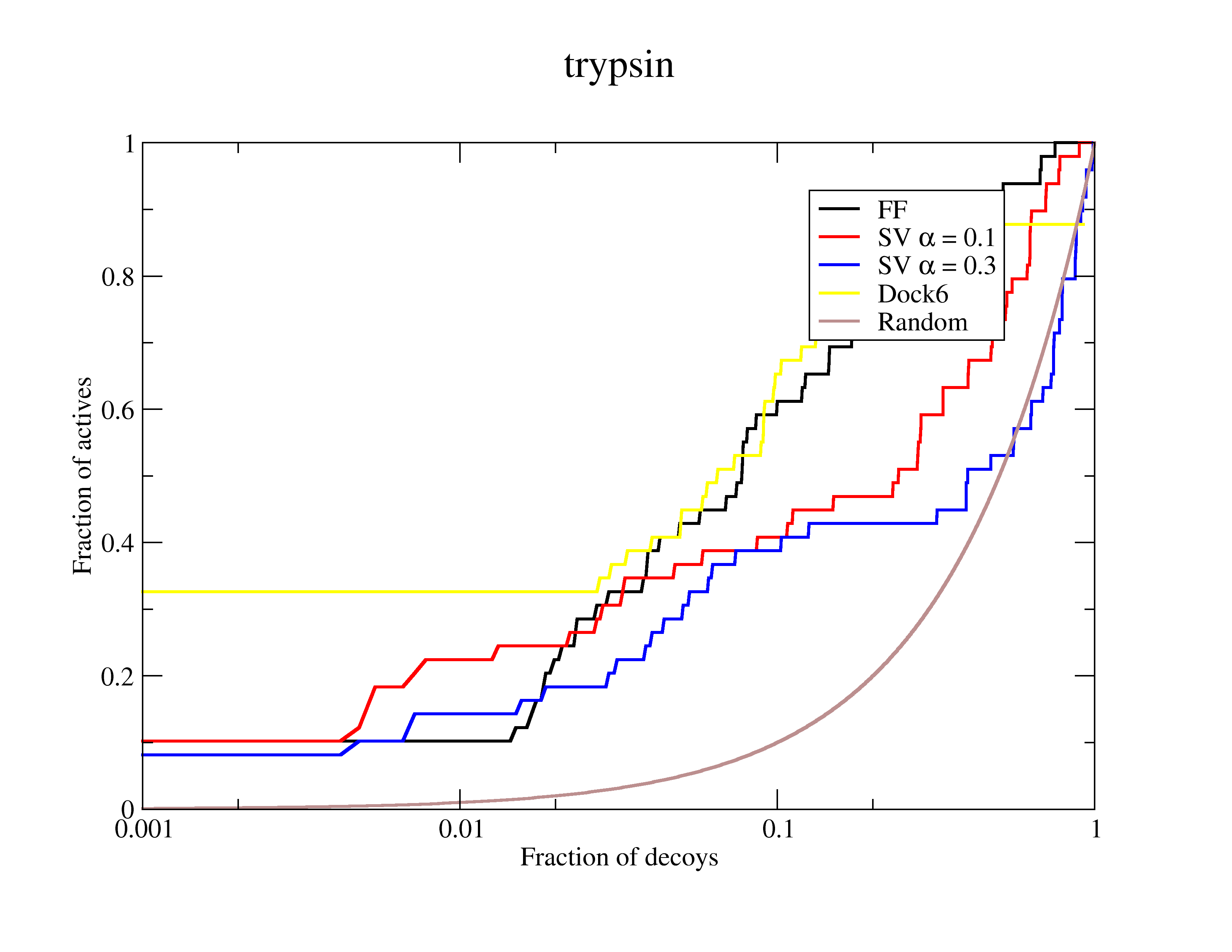

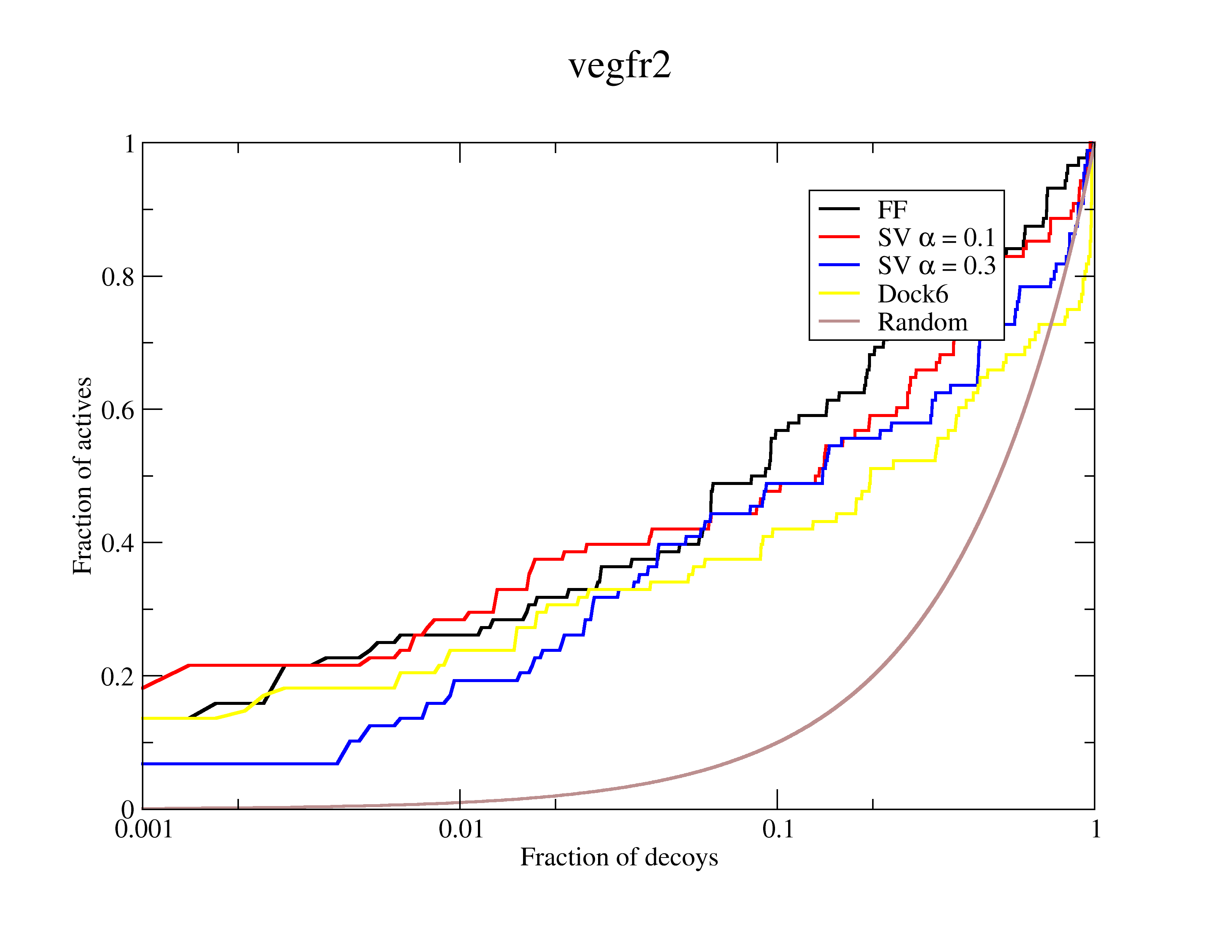

Supplement: S1 File — The brown line shows the enrichment expected to for a random distribution of ligands and decoys. The enrichments obtained with FF (black line), SV (α = 0.1 kcal.mol-1.e-2, red), SV (α = 0.3 kcal.mol-1.e-2, blue) and DOCK6 (yellow) are shown. (DOCX) [file pone.0174336.s001.docx]
